# Supplementary material for: Infection and biogeographical characteristics of Paragonimus westermani and P. skrjabini in humans and animal hosts in China: A systematic review and meta-analysis
Source: PLoS Negl Trop Dis. 2024 Aug 5;18(8):e0012366. doi: 10.1371/journal.pntd.0012366 (PMC11326572; doi:10.1371/journal.pntd.0012366)
Supplement: S1 Fig — (a) Paragonimus in humans; (b) P. westermani in the first intermediate host; (c) P. skrjabini in the first intermediate host; (d) P. westermani in the second intermediate host; (e) P. skrjabini in the second intermediate host; (f) P. westermani in animal reservoir; (g) P. skrjabini in animal reservoir. (DOC) [file pntd.0012366.s012.doc]

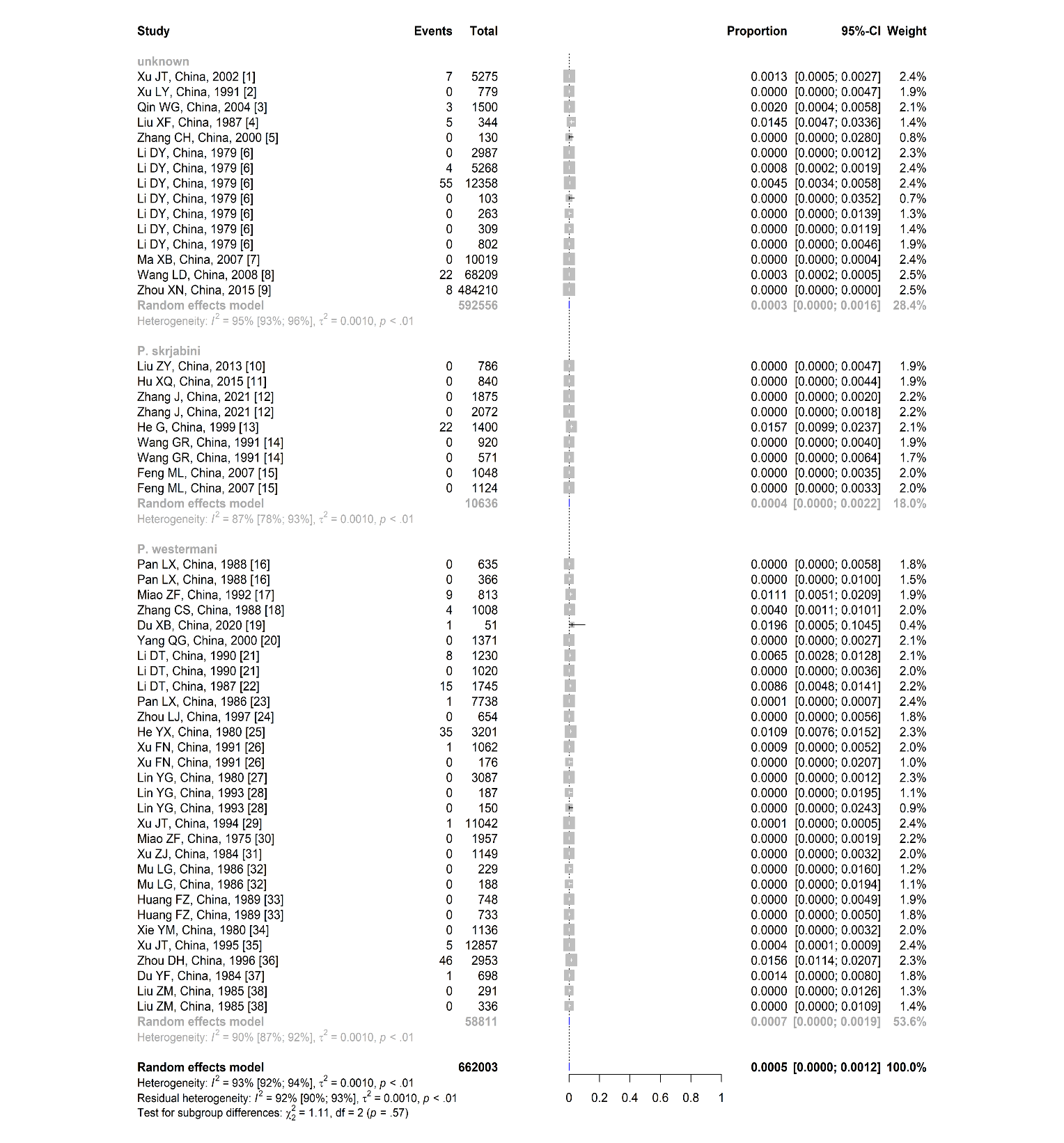
S1A_Fig


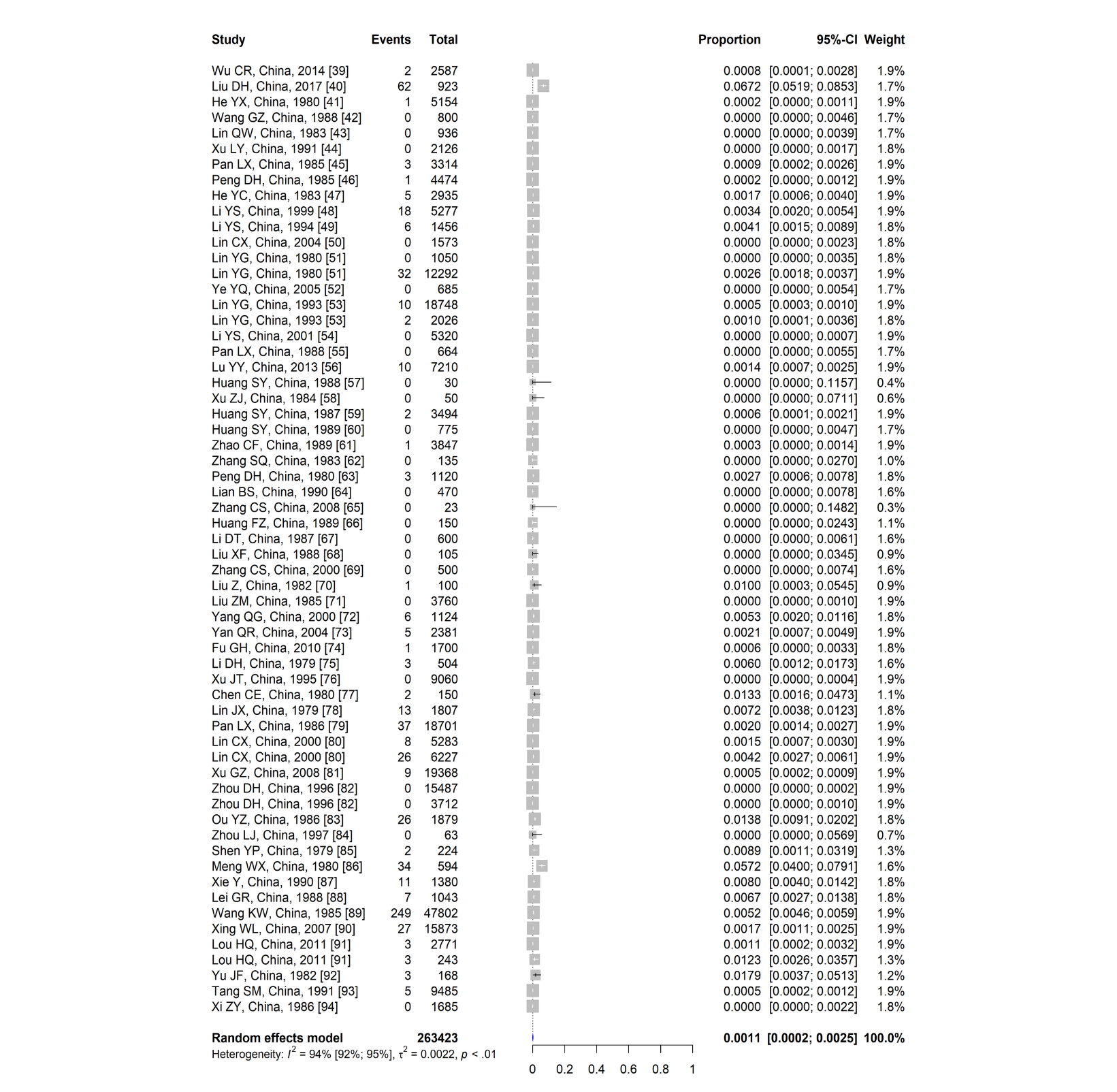
S1B_Fig


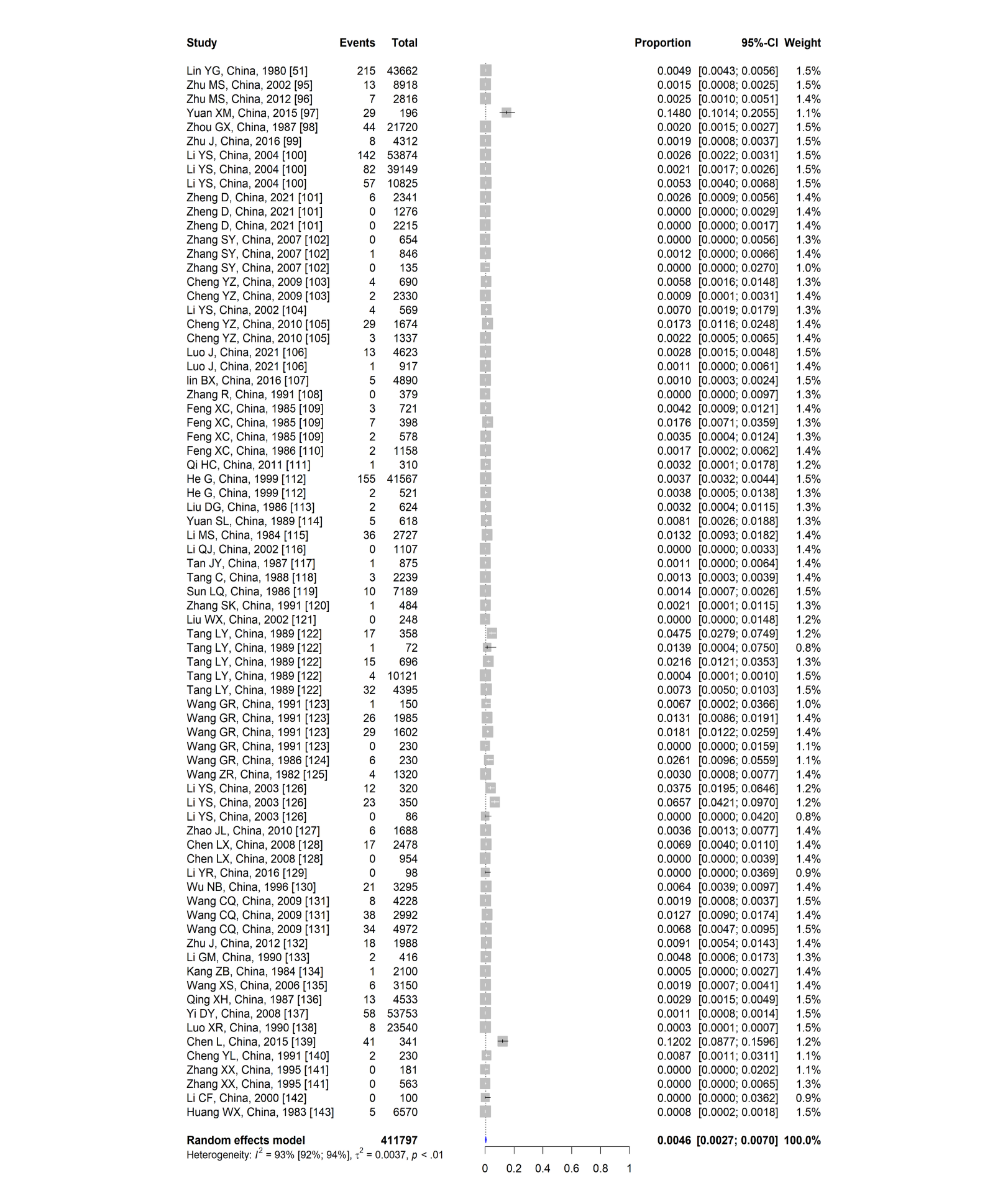
S1C_Fig


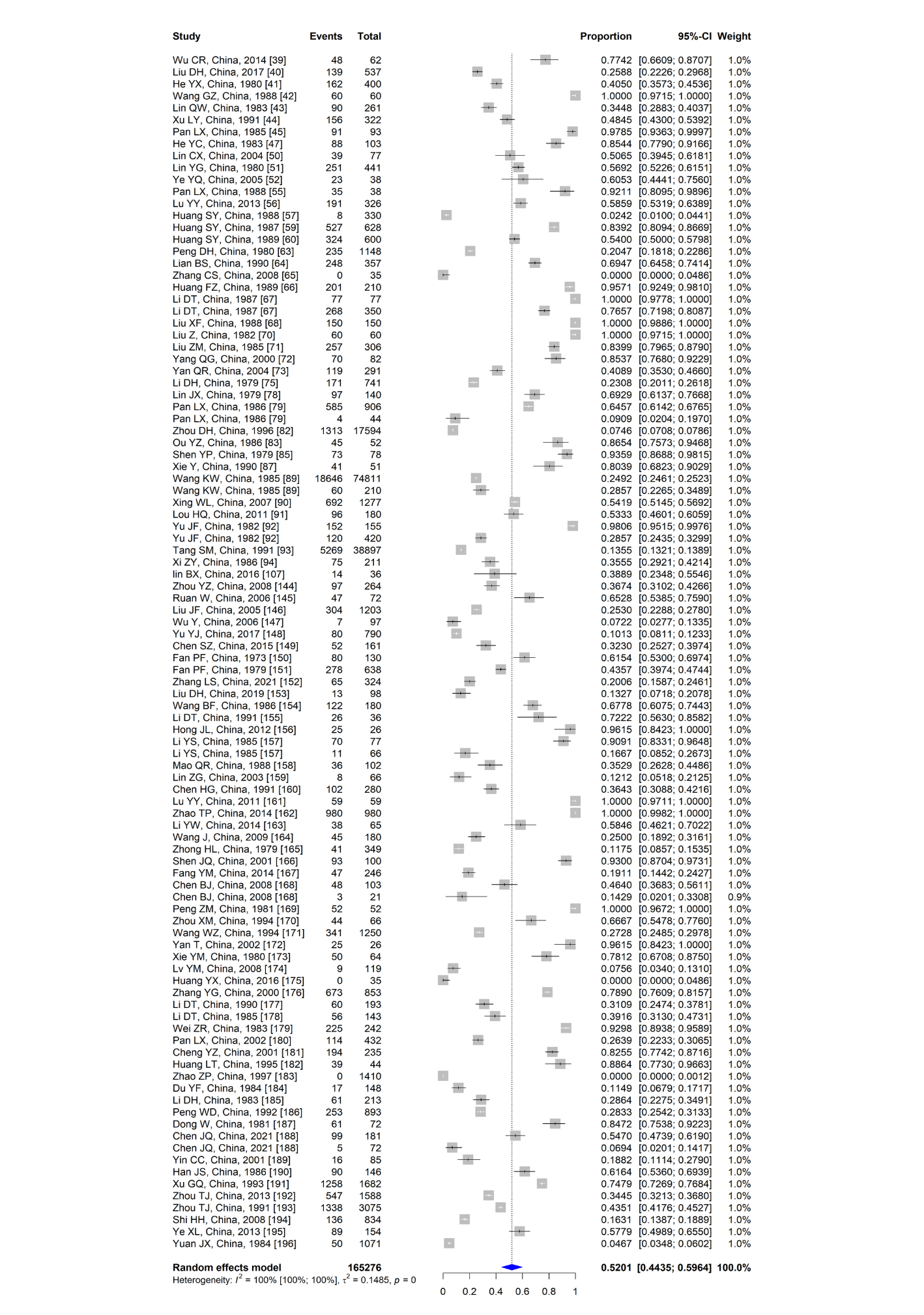
S1D_Fig


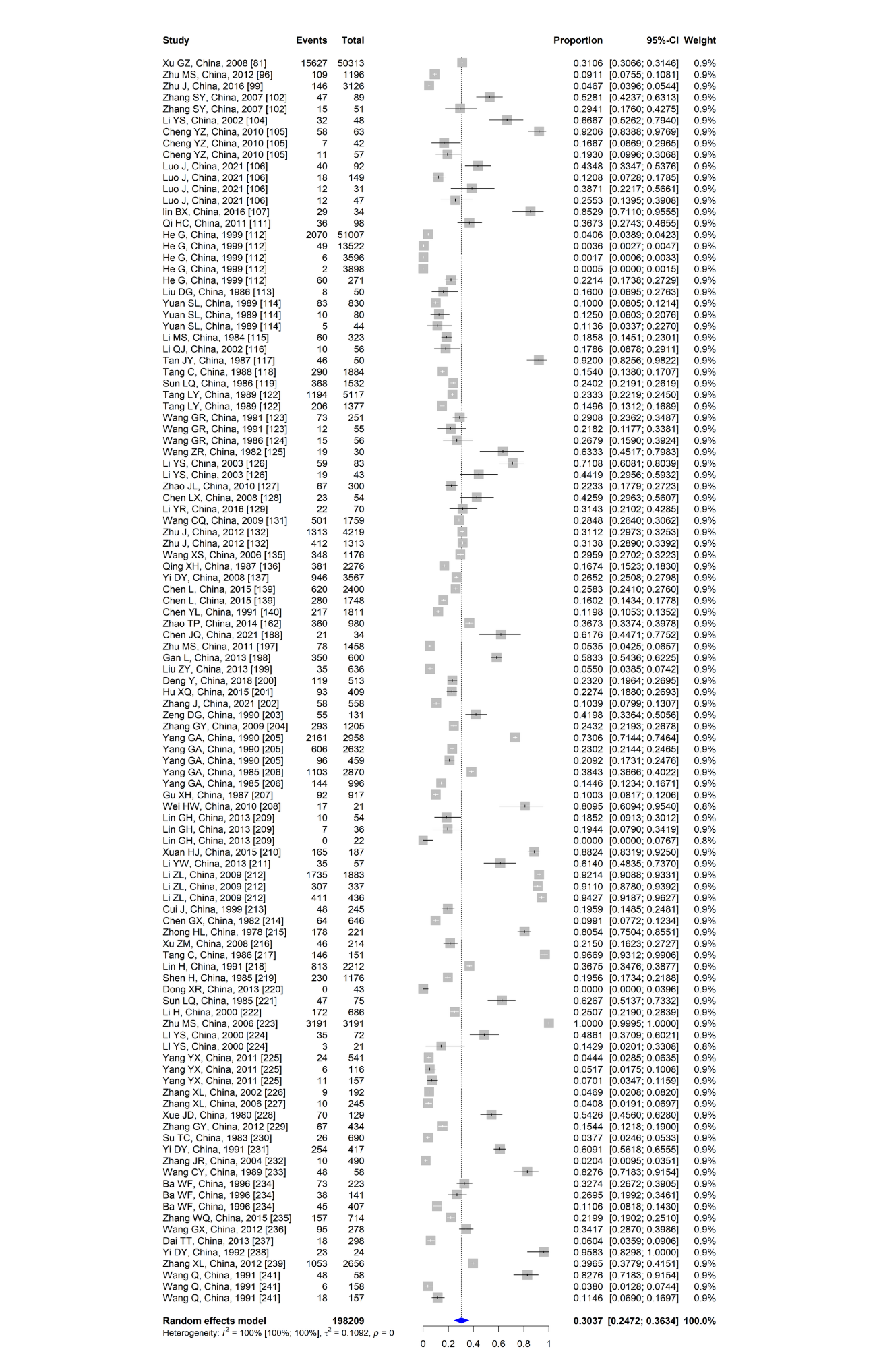
S1E_Fig


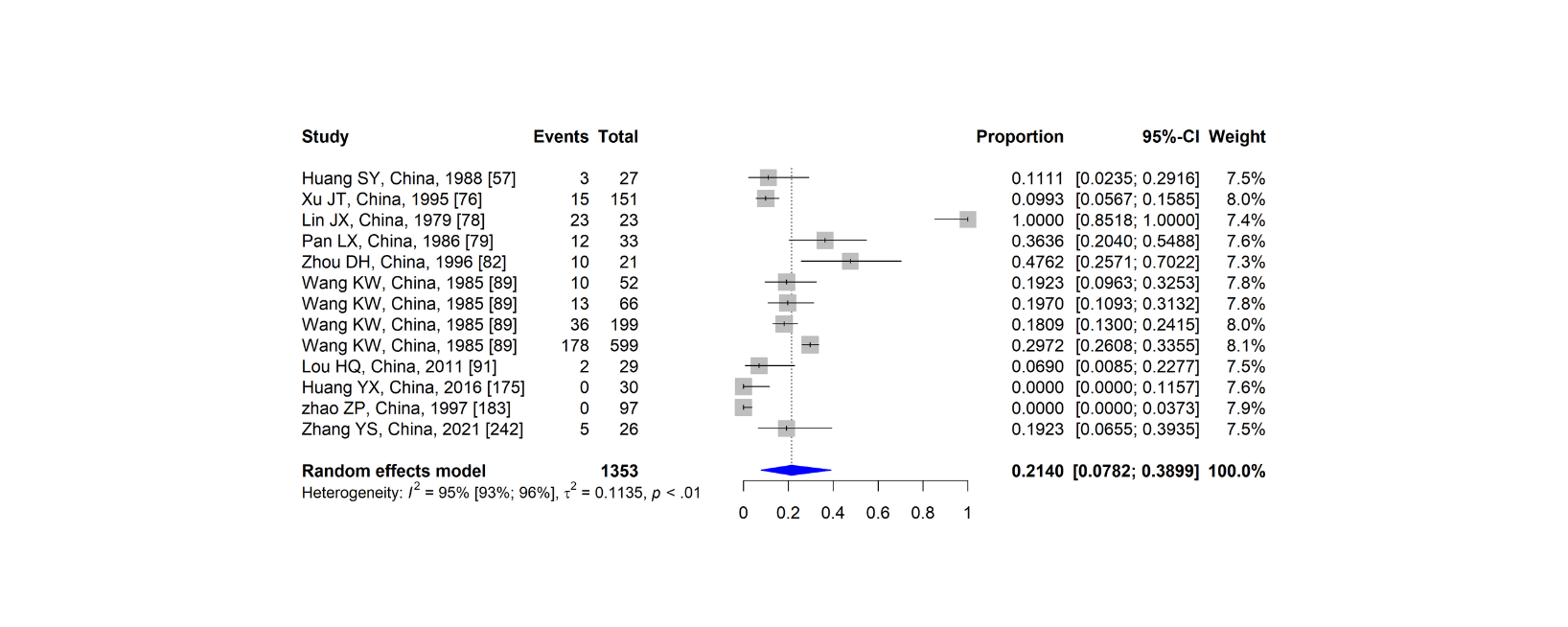

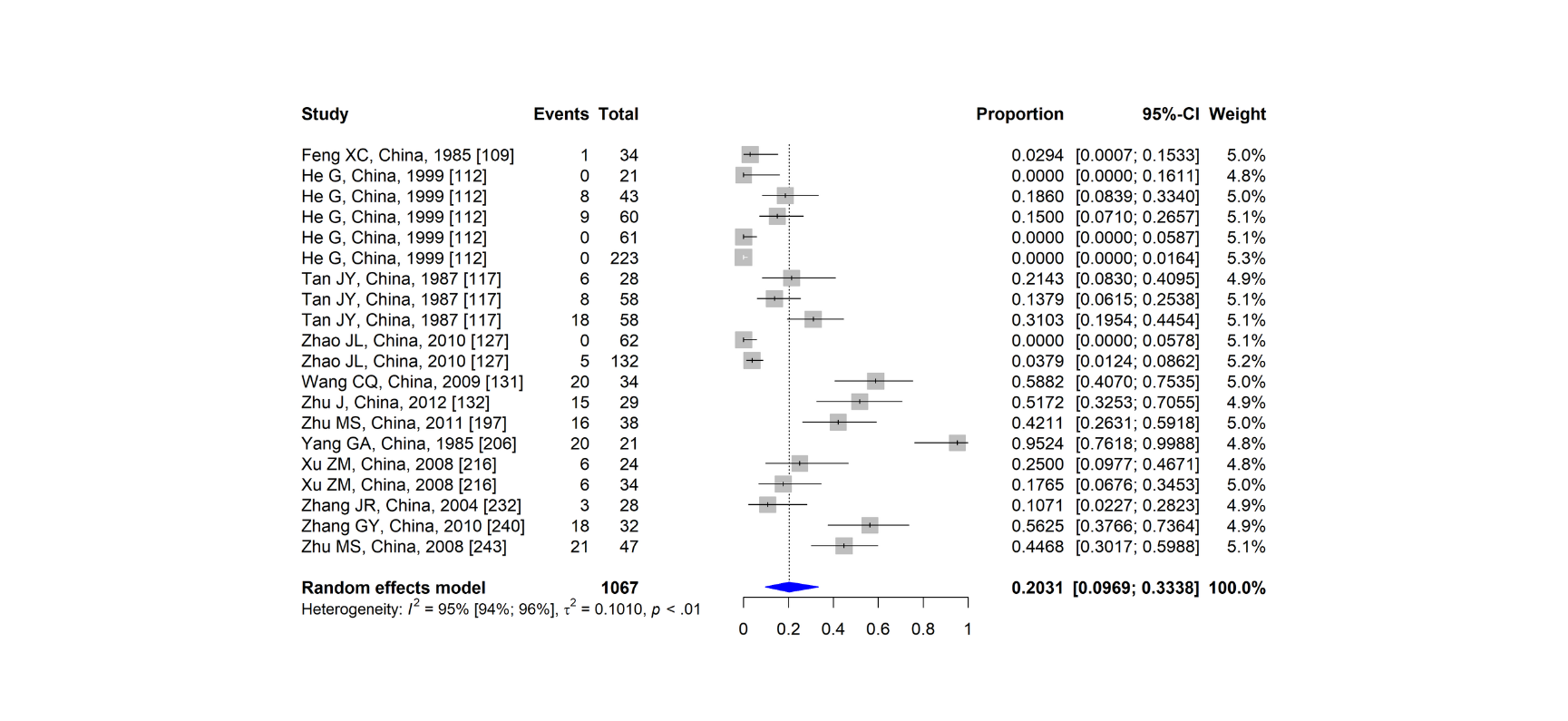
S1F_Fig

S1G_Fig

**References**

1. Xu JT, Mao LL. Monitoring and Analysis of Pulmonary Fluke Disease in Liaoning Province from 1990 to 2001. J. Pathog. 2002, (05):71. Chinese.

2. Xu LY, Zhang YK, Xu FH, Xie HL, Zhu ZH, Shao JQ, et al. Epidemiological Investigation Report on Pulmonary Fluke in Changshan County. Chin Prev Med J. 1991,(05):22-23. Chinese.

3. Tan WG, Tang LY, Bai Y. Current Status Investigation and Analysis of Pulmonary Fluke Disease in Chengkou County. Proceedings of the 2004 Academic Exchange Conference of the Chongqing Preventive Medicine Association. 83. 5013888888889.Chinese.

4. Liu XF, Wang GW, Huang SY, Wen JS. Epidemiological Investigation of Pulmonary Fluke Disease in Yangzi Shao Town, Huinan County, Jilin Province. Jilin Med J. 1988,(06):378. Chinese.

5. Zhang CS. Investigation on the Prevalence of Pulmonary Fluke Disease in Piaoriver Town, Jiaohe City, Jilin Province. Chin J. Zoon. 2000,(05):107. Chinese.

6. Li DY, Wang CX, Li WF, Zhu YS. Investigation Report on the Prevalence of Pulmonary Fluke Disease in Liaoning Province. J Chin Med Univ. 1979,(02):41-45. Chinese.

7. Ma XB, Cai L, Zhang BX, Fu YH, Chen J, Cao L, et al. Serological Epidemiological Investigation of Pulmonary Fluke Disease in the Population of Shanghai City. Chin J Zoonoses. 2007,(03):303-304. Chinese.

8. Wang LD, Qi XQ, Wang Y. Report on National Survey of Important Human Parasitic Disease in China / Prepared by the Department of Disease Control and Prevention, Ministry of Health: People's Medical Publishing House2008.

9. Zhou XN, LI SZ, LI ZJ, Chen YD. Report on National Survey of Important Human Parasitic Disease in China (2015) / Prepared by the National Institute of Parasitic Diseases, Chinese Center for Disease Control and Prevention: People's Medical Publishing House2018.

10. Liu ZY, Liu CF, Li BX, Tang DH, Li M, Yuan XM, et al. Survey on the Epidemics of Paragonimiasis, Kaijiang County,2012. Prev. Med. Trib. 2013,19(04):255-57+260. Chinese.

11. Hu XQ, Zhao Q, Liu ZX, Gui GY, Peng HW, Du MR. Epidemiological Survey on Paragonimiasis Situation in Dazhou,2013. J. Prev. Med. Info. 2015,31(02):136-39. Chinese.

12. Zhang J, Xia J, Zhang HX, Zhu H, Wu DN, Wan L, et al. Surveillance on paragonimiasis infection in Hubei Province from 2018 to 2020. Chin J Parasitol Parasit Dis. 2021,39(05):600-605. Chinese.

13. He Gang, Wei MB, Li SL, Zhu QY. Investigation on natural foci of paragonimiasis in Guangxi. Chin J Zoonoses. 1999,15(6):45-48. Chinese.

14. Wang GR, Cao GH, Cao JS, Liang XS. Epidemiological investigation of paragonimiasis in Loudi District of Hunan Province. Chin J Parasitol Parasit Dis. 1991,(04):291-92. Chinese.

15. Feng ML, Shang YL, Yan XC. Epidemiological investigation of paragonimiasis in jinmiaopu Town, Zezhou County, Shanxi Province. 2007 National Conference on Cancer Epidemiology and Etiology. 2007:131-33. Chinese.

16. Pan LX, Li CP, Yang ZH, Yang YH, Huang FS, Guo DG, et al. Epidemiological investigation of paragonimiasis in Taoshi village, Maoping, Dapu County, Guangdong Province South China. J Prev Med. 1988,(01):45-46. Chinese.

17. Miao ZF, Li YB, Zhu X, Ren PS, Li CY, Li XS. *Paragonimus westermani* found in Hebei Province. J. Pathog. 1992,(04):309. Chinese.

18. Zhang CS, Yuan JH. Type and epidemiological investigation of *paragonimiasis westermani* in Huadian City, Jilin Province. J. Pathog. 1988,(S1):97. Chinese.

19. Du XB, Wang JP, Wang XW, Zheng W, Deng Y, Chen WQ, et al. Disposal and focus investigation of a case of paragonimiasis in Jiyuan City. Mod Prev Med. 2020,31(07):553-55. Chinese.

20. Yang QG, Liao J, Liu HC, Peng JG. Investigation report on paragonimiasis in Wugong Mountain Area of Anfu County, Jiangxi Province. J Jgs Med Col. 2000,(04):74-76. Chinese.

21. Li DY, Zhou JS, Liu TC, Tan DK, Tan QW, Li XC, et al. Discovery and difference of two types of *paragonimiasis westermani* in Fengcheng County, Liaoning Province. J Chin Med Univ. 1990,(02):88-91+99. Chinese.

22. Li DY, Yuan JH, Ma YX, Hu ZG, Zhou HY. Recent epidemic situation of paragonimiasis in Kuandian County, Liaoning Province. Chin J Parasitol Parasit Dis. 1987,(04):72. Chinese.

23. Pan LX, Lin XB, Yang ZH, Xie ZC, Xie YQ, Qiu MS. Discovery of paragonimiasis epidemic area in Meixian County, South China. J Prev Med. 1986,(01):89-92. Chinese.

24. Zhou LJ. Epidemiological investigation of paragonimiasis in Rucheng County. Prac Prev Med. 1997,(04):253. Chinese.

25. He YX, Huang DS, Hu YQ, Zhu XH, Zhang YN. Study on epidemiology and pathogenic biology of paragonimiasis in Fanchang County, Anhui Province. Acta Acad Med Sin. 1980,(01):57-63+79. Chinese.

26. Xu FN, Wu WD, Guo JD, Hu WF, Wang ZH, Mei JD, et al. Distribution of human parasitic flukes in the Yangtze River Valley of Anhui Province. Chin J Parasitol Parasit Dis. 1991,(S1):37-40. Chinese.

27. Lin YG, Kang J, Lv JH, Lin MX, He YC, Chen QQ, et al. Discovery and etiology of paragonimiasis epidemic area in Jianou County, Fujian Province. Curr Zool. 1980,(01):52-60+108. Chinese.

28. Lin YG, Yang WC, Yan RL, Hong LX, He YC, Yang CC, Discovery and etiology of paragonimiasis epidemic area in Jianou County, Fujian Province. Wy Sci J. 1993,(00):55-63. Chinese.

29. Xu JT, Qian ZF, Wang L, Xiao WH, Wang JQ, Luan JY, et al. Study on eliminating paragonimiasis in the water diversion project area of the Fuer River. China Public Health J. 1994,(10):450-51. Chinese.

30. Miao ZF. Preliminary investigation on paragonimiasis in Yiyang County, Henan Province. Mod Prev Med. 1975,(02):34-39. Chinese.

31. Xu ZJ, Huang SY. Preliminary epidemiological investigation of paragonimiasis in Pingshan boiler plant of Heilongjiang Province. J Harbin Med Univ. 1984,(02):81-84. Chinese.

32. Liao CG. Epidemiological investigation and control of paragonimiasis westermani in Daye, Huangshi City. China Public Health J. 1986,(01):61-62. Chinese.

33. Huang FZ, Cui CQ, Quan FS. Epidemiological investigation of paragonimiasis in Antu County, Jilin Province. J Med Sci YB Univ. 1989,(01):8-11. Chinese.

34. Xie ZM, Wu FD, Huang WC, Zhou XM. Investigation on paragonimiasis in Sandu District, Tonggu County, Jiangxi Province. Jx Med J. 1980,(05):52-53. Chinese.

35. Xu JT, Qian ZF, Xiao WH, Wang L, Sun T, Wang JQ, et al. Epidemiological investigation of paragonimiasis in Xinbin County, Liaoning Province. J. Pathog. 1995,(01):63. Chinese.

36. Zhou DH, Li ZH. Investigation on paragonimiasis in Ninghai County. Chin J Zoonoses. 1996,(05):57-58. Chinese.

37. Du YF, Hu SL. An epidemic area of paragonimiasis was first found in Shangyu county. Zhejiang M J. 1984,(01):60. Chinese.

38. Liu ZM, Bai GF, Lian JA, Xu JW, Li RH, Xu FW, et al. Investigation on the prevalence of paragonimiasis in Jiangnan township of Jilin City. J Jl Univ. 1985,(02):221-26. Chinese.

39. Wu CR, Chen BC, Lin CX. Investigation on Paragonimus Foci in Ninghua County in 2012. Prev Med Trib. 2014,20(02):130-31. Chinese.

40. Liu DH, Guo JD, Wang TP, Zhang SQ, Zhu L, Jin W, et al. Analysis of monitoring results of paragonimiasis in Anhui from 2013 to 2016. J Tro Dis Para. 2017,15(3):155-58Chinese.

41. He YX, Huang DS, Hu YQ, Zhu XH, Zhang YN. Epidemiology and Pathogenic Biology of paragonimiasis in Fanchang County, Anhui Province. Acta Acad Med Sin. 1980,(01):57-63+79. Chinese.

42. Wang GZ, Zhang WY, Zhu LX, Qi ZC, Li ZX, Zhao FQ, et al. Investigation on the infection of Intermediate host of paragonimus in Xiaocheng Village, Baoqing County. HLJ Med Pha. 1988,(02):107-108+187. Chinese.

43. Lin QW, Ma LY, Chen DQ, Yang ZC. Investigation on infection of paragonimus, its Intermediate host and population in Chashan commune. J W Med Univ. 1983,(02):48-53. Chinese.

44. Xu LY, Zhang YK, Xu FH, Xie HL, Zhu ZH, Shao JQ, et al. Epidemiological investigation of paragonimus in Changshan County. Chin J Prev Med. 1991,(05):22-23. Chinese.

45. Pan LX, Qiu MS, Yang YH. Investigation on paragonimiasis in Fengxi Forest Farm of Dabu County. Chin J Prev Med. 1985,19(2):122. Chinese.

46. Peng DH, Zhou Y, Zhou SL, Liao LG, Guo TQ, Ai DH, et al. Observation on the Larvae of *Paragonimus westermani* in Eastern Hubei. Chin J Parasitol Parasit Dis. 1985,(01):64. Chinese.

47. He YC, Lin YG, Hong LX, Yang WC, Bi WD, Liu GH. Investigation on the epidemiology of paragonimiasis in Jiangle County, Fujian Province. J Xm Univ. 1983,(01):102-109. Chinese.

48. Li YS, Lin JX, Cheng YZ, Lin CX. Changes in the infection rate and its causes in the infected areas of schistosomiasis in Fujian Province. J. Pathog. 1999,(04):44-46. Chinese.

49. Li YS, Lin JX. Investigation on the etiology of paragonimiasis in Minhou County, Fujian Province. Wy Sci J. 1994,(00):162-66. Chinese.

50. Lin CX, Yang XD, Li LS, Liao YJ, Cheng YZ, Chen YW, et al. Investigation on the first and second Intermediate host of paragonimus in Youxi County. Strait J Prevent Med. 2004,(05):31. Chinese.

51. Lin YG, Kang J, Lv JH, Lin MX, He YC, Chen QQ, et al. Discovery and Etiological Study on the Epidemic Area of Paragonimus in Jianou County, Fujian Province. Curr Zool. 1980,(01):52-60+108. Chinese.

52. Ye YQ, Xia YJ. Investigation on Intermediate host of paragonimus in Yangmeizhou Village, Kengdi Township, Shouning County, Fujian Province. J Tro Dis Para. 2005,(01):32. Chinese.

53. Lin YG, Yang WC, Yan RL, Hong LX, He YC, Yang CC, Etiological and Epidemiological Investigation of Pulmonary Schistosomiasis in Zhangzhou, Fujian Province Wy Sci J. 1993,(00):55-63. Chinese.

54. Li YS, Cheng YZ, Chen BJ, Jiang WM, Jiang JS, Shen JJ, et al. Preliminary investigation on the etiology of paragonimiasis in Guangze County. Para Infect Dis. 2001,(02):71-72+98. Chinese.

55. Pan LX, Li CP, Yang ZH, Yang YH, Huang FS, Guo DS, et al. Epidemiological survey of paragonimiasis in Taoshi Village, Maoping County, Dabu County, Guangdong Province. GD J H Epi Prev. 1988,(01):45-46. Chinese.

56. Lu YY, Liu Q, Tang GX, Shen HX, Zhong JX, Xie QC, Fu GHD istribution and DNA Sequence Analysis of *Paragonimus westermani* in Some Regions of Guangdong Province. Chin J Schi Ctl. 2013,25(03):275-79+283. Chinese.

57. Huang SY, Fan SQ, Zhao SY, Chen Y, Xu ZJ, Liu GJ, et al. Discovery of Parthenogenetic *Paragonimus westermani* in Aihui County, Heilongjiang Province. Chin J Parasitol Parasit Dis. 1988,(02):63. Chinese.

58. Xu ZJ, Huang SY. Preliminary Epidemiological Investigation on paragonimiasis at Pingshan Boiler Factory in Heilongjiang Province. J Harbin Med Univ. 1984,(02):81-84. Chinese.

59. Huang SY, Fan SQ, Sun XQ, Zhao SY, Zhao QF, Liu GJ, et al. Biological investigation of the pathogen of paragonimiasis in the Suifenhe River Basin, Heilongjiang Province. Chin J Zoonoses. 1987,(01):40-42. Chinese.

60. Huang SY, Fan SQ, Zhao JB. Investigation on the Pathogen of paragonimiasis in Shahezi Town, Wuchang County, Heilongjiang Province. J Harbin Med Univ. 1989,(06):452-53. Chinese.

61. Zhao CF, Yang SJ, Xue J. Natural Focus of Paragonimus in Dangyang County, Hubei Province. Chin J Zoonoses. 1989,(04):57-58. Chinese.

62. Zhang SQ, Jiang RX, Yang LT, Xie NF. A report on the pathogen of *Paragonimus westermani* in hubei province. Chin J Zool. 1983,(02):20-21. Chinese.

63. Peng DH, Tang C, Xiang XS. Investigation report on paragonimiasis in Wufeng County, Hubei Province. Med J WH Univ. 1980,(02):29-32. Chinese.

64. Lian BS, Dai DL, Chen XG, Zhao HG, Kong HB, Wan ZY. Preliminary investigation of paragonimiasis in Xianning, Hubei. J. Pathog. 1990,(02):146. Chinese.

65. Zhang CS, Liu HJ, Yu JZ, Zhang YB, Li YJ Investigation on the epidemic status of paragonimiasis in Huadian City. J. Pathog. 2008,(01):42+38. Chinese.

66. Huang FZ, Cui CQ, Quan FS. Epidemiological survey of paragonimiasis in Antu County, Jilin Province. J Med Sci YB Univ. 1989,(01):8-11. Chinese.

67. Li DY, Kong QC, Wang JC, Zhang CS, Zhang DS, Liu YZ. Epidemiological investigation of paragonimiasis in Hongshi Township, Huadian County, Jilin Province. Chin J Zoonoses. 1987,(03):35-37. Chinese.

68. Liu XF, Wang GW, Huang SY, Wen JS. Epidemiological survey of paragonimiasis in Xiangshao Town, Huinan County, Jilin Province. JL Med J. 1988,(06):378. Chinese.

69. Zhang CS. Investigation on the prevalence of paragonimiasis in Piaohe Town, Jiaohe City, Jilin Provinc. Chin J Zoonoses. 2000,(05):107. Chinese.

70. Liu Z, Chen MS, Li L. Epidemiological factors of paragonimiasis in Jingyu County, Jilin Province. J Jl Univ. 1982,(02):100-104. Chinese.

71. Liu ZM, Bai GF, Lian JA, Xu JW, Li RH, Xu FW, et al. Investigation on the prevalence of paragonimiasis in Jiangnan Township, Jilin City. J Jl Univ. 1985,(02):221-26. Chinese.

72. Yang QG, Liao J, Liu HC, Peng JG. Investigation report on paragonimiasis in Wugong mountain area of Anfu County, Jiangxi Province. J Jgs Med Col. 2000,(04):74-76. Chinese.

73. Yan QR, Yan T, Zhou XM, Li YS, Zhu CC, Shi LB, et al. Epidemiological investigation of paragonimiasis in Jiangxi Province. Chin J Parasitol Parasit Dis. 2004,(04):58-60. Chinese.

74. Fu GH, Deng WQ, Lu YY, Shen HX, Li HG, et al. Report on a newly discovered epidemic focus of *Paragonimus westermani* in Lechang. J TROP MED. 2010,10(08):1020-22. Chinese.

75. Li DY, Wang CX, Li WF, Zhu YS. A survey report on the prevalence of paragonimiasis in Liaoning Province. J Chin Med Univ. 1979,(02):41-45. Chinese.

76. Xu JT, Qian ZF, Xiao WH, Wang L, Sun T, Wang JQ, et al. Epidemiological investigation report on paragonimiasis in Xinbin County, Liaoning Province. J. Pathog. 1995,(01):63. Chinese.

77. Chen CE, Chen JR, Guo SX, Xiong ZH, Bi K. Preliminary report on the investigation of the prevalence of paragonimiasis in Baisha Commune, Liuyang County. J CENT SOUTH UNIV. 1980,(04):302-303. Chinese.

78. Lin JX, Li YS, Wu ZY. Epidemiological investigation report on paragonimiasis in Longhu logging farm. Chin J Zool. 1979,(03):18-21. Chinese.

79. Pan LX, Lin XB, Yang ZH, Xie ZC, Xie YQ, Qiu MS. Discovery of the endemic area of paragonimiasis in Meixian area South China. J Prev Med. 1986,(01):89-92. Chinese.

80. Lin CX, Li YS, Cheng YZ, Lu CJ, Ye XP, Wu JY. Comparative study on the paragonimus cercariae and their similar cercariae in the bodies of Oncomelania hupensis and Erhai snails. Strait J Prevent Med. 2000,(06):11-12. Chinese.

81. Xu GZ, Qian BZ, Ye LP, Zhang JN, Lu F, Sun YW. Epidemic status of paragonimiasis in Ningbo City. Chin J Parasitol Parasit Dis. 2008,26(06):449-51. Chinese.

82. Zhou DH, Li ZH. Investigation on paragonimiasis in Ninghai County. Chin J Zoonoses. 1996,(05):57-58. Chinese.

83. Ou YZ, Yao LZ, Yao X. Epidemiological investigation of paragonimiasis in Renju District, Pingyuan County South China. J Prev Med. . 1986,(01):93-94. Chinese.

84. Zhou LJ. Epidemiological survey of paragonimiasis in Rucheng County. Prac Prev Med. 1997,(04):253. Chinese.

85. Shen YP. A new record of the degree of infection of paragonimid larvae in stone crabs. Jiangsu Med J. 1979,(10):46. Chinese.

86. Meng WX, Zhou SF, Hong WC, Li LZ, Lei CQ, Shou GC, et al. Preliminary investigation on the prevalence of paragonimiasis in Suichang County and the discovery of paragonimiasis in Sanping County. Zhejiang M J. 1980,(01):24-28+51. Chinese.

87. Xie Y, Xu GQ, Peng XM, Lu HY, Hu YG. Investigation on the prevalence of *paragonimiasis westermani* in Huanggang area of Yifeng County. J. Pathog. 1992,(03):235-36. Chinese.

88. Lei GR, Ye CC. Preliminary Investigation on the Host and Population Infection of Paragonimus in Yunhe and Jingning Counties. Chin J Zoonoses. 1988,(05):62. Chinese.

89. Wang KW, Shen HM. Investigation on the prevalence of paragonimiasis in Zhejiang Province. Chin J Parasitol Parasit Dis. 1985,(01):59+79. Chinese.

90. Xing WL, Chen XD, Xie KX, Zou SW, Yao ZD, Liu QZ, et al. Discovery of a medium high focus of paragonimiasis in Taishun County, Zhejiang Province. Chin J Zoonoses. 2007,(06):614-16. Chinese.

91. Lou HQ, Hu Y, Jin YJ, Yu XT, Wang L, He XY, et al. Investigation on the Natural reservoir of paragonimus and identification of its species in Jinhua, Zhejiang Province. J. Pathog. 2011,29(05):348-52. Chinese.

92. Yu JF, Shen ZQ. Epidemiological investigation report on paragonimiasis in Pangshan area, Zhejiang Province. Chin J Zool. 1982,(02):40-41. Chinese.

93. Tang SM, Xu YY, Ye LP, Zhou DH. Investigation on the prevalence of paragonimiasis in Ningbo City, Zhejiang Province. J. Pathog. 1991,(01):74. Chinese.

94. Xi ZY, Huang MY, Rong YL, Deng BJ, Weng Y, Zhang JG, et al. Preliminary investigation of paragonimiasis in mountain villages of Shouxian County, Zhejiang Province. Fudan Univ J Med Sci. 1986,(01):53-56. Chinese.

95. Zhu MS, Zhu YX, Liu Q, Liu WX, Bei T, Chang XD, et al. Investigation on the distribution of natural foci of paragonimiasis in Shiyan area. J. Pathog. 2002,(03):36+44. Chinese.

96. Zhu MS, Zhu J, Wei RH, Zhu YX. Analysis on the changes and causes of the epidemic situation of *Paragonimus skrjabini* in Shiyan, Hubei Province. J Shanxi Univ Med. 2012,43(12):921-23. Chinese.

97. Yuan XM, Liu ZY, Liu CF, Li BX, Lin RJ, Tang DH, et al. Epidemiological survey of paragonimiasis in Kaijiang County in 2013. Mod Prev Med. 2015,42(09):1555-58. Chinese.

98. Zhou GX, Luo XR, Xu XM. Epidemiological investigation of paragonimiasis in Daxian and Wanyuan areas. SC Med J. 1987,(02):104-105. Chinese.

99. Zhu J, Wei RH, Liu DD, Yang SG. Analysis on the changes and causes of infection of Intermediate host of *Pagumogonimus skrjabini* in northwest Hubei. J HB Univ Med. 2016,35(01):12-14. Chinese.

100. Li YS, Cao SP, Zhang SY, Zhou XM, Xu LS, Lin JX. Investigation on the species, distribution and infection rate of the first Intermediate host of paragonimus in Fujian Province. Strait J Prevent Med. 2004,(06):1-3. Chinese.

101. Zheng D, Lin CX, Cai WW, Xie HG. Investigation on the infection of the first Intermediate host of Paragonimus and its cercariae in Pingnan County, Fujian Province. J Tro Dis Para. 2021,19(05):266-69. Chinese.

102. Zhang SY, Xu LS, Li YS, Lin CX, Cheng YZ, Zhou XM, et al. Epidemiological Investigation of Paragonimus in Sanyuan District, Sanming, Fujian Province. Strait J Prevent Med. 2007,(04):10-12. Chinese.

103. Cheng YZ. New epidemic area and transmission host of *Paragonimus skrjabini* in Fujian Province. NA. 2014Chinese.

104. Li YS, Zhou AP, Zhou XM, Cheng YZ, Zhu HH, Lin CX, et al. Investigation on the etiology of paragonimiasis in Songxi County, Fujian Province. J TROP MED. 2002,(03):245-48+2Chinese.

105. Cheng YZ, Li LS, Lin GH, Zhou PC, Jiang DW, Jiang DW, et al. Investigation of Paragonimiasis Foci in Youxi, Yongtai and Pinghe County, Fujian Province. Chin J Parasitol Parasit Dis. 2010,28(06):406-10. Chinese.

106. Luo Y, Chen JQ, Jiang DW, Zhou PQ, Cai MS, Cheng YZ. Population and Infection of Paragonimus Intermediate host in Southeast Youxi County, Fujian Province. Chin J Parasitol Parasit Dis. 2021,39(05):646-51. Chinese.

107. Lin BX, Wei HW, Li YS, Wu SQ, Kong FZ, Fan YJ, et al. Investigation of Paragonimiasis Foci in the East of Zhenghe County, Fujian Province Chin J Schi Ctl. 2016,28(04):418-21. Chinese.

108. Zhang R. Title: Comprehensive report on epidemiological investigation of paragonimiasis in various regions (subtitle: investigation of *paragonimiasis skrjabini* in Nanzhang County, Hubei Province). Chin J Parasitol Parasit Dis. 1991, (3):79-80. Chinese.

109. Feng XC. Epidemiological survey of paragonimiasis in Guzhang County. Chin J P H. 1985,(02):38-39. Chinese.

110. Feng XC, Zhang KR, Zhou WH, Chen SZ, Li ZJ, Feng XC, et al. The first Intermediate host of paragonimus was found again in Guzhang County. One new snail species, Qiuji and Glide Oncomelania. Chin J P H. 1986,5(05):38-39. Chinese.

111. Kuang HC, Lu YY, Shen HX. First Report on the Focal Area of Lutian's Pagumogonimus in Conghua City, Guangdong Province. Chin J Mod Med. 2011,21(07):766-68+772. Chinese.

112. He Gang, Wei MB, Li SL, Zhu QY. Investigation on Natural Focal Areas of Paragonimus in Guangxi Province. Chin J Zoonoses. 1999,15(6):45-48. Chinese.

113. Liu DG, Nong ZJ. Preliminary investigation of paragonimiasis in Xing'an, Guangxi. Chin J Parasitol Parasit Dis. 1986,(03):75-76. Chinese.

114. Yuan SL, Li MG. . Investigation and study on paragonimiasis in Chishui County, Guizhou Province. Chin J Zool. 1989,(01):5-7. Chinese.

115. Li MG, Cui SB, Chen ZY, Wang BB, Hu QX. Epidemiological investigation of paragonimiasis in Kaiyang, Wanshan, and Jiangkou counties of Guizhou Province. Chin J Parasitol Parasit Dis. 1984,(01):57. Chinese.

116. Li QJ, Zhao YH, Zhang RJ, Gao GB. Epidemiological investigation of paragonimiasis in Yuqing County, guizhou province. J Med P Ctl. 2002,(11):627-28. Chinese.

117. NA Investigation on the prevalence of paragonimiasis in Lichuan, Hubei Province, Bazhong, Sichuan Province, and Yushan, Jiangxi Province. Chin J Parasitol Parasit Dis. 1987,(04):65. Chinese.

118. Tang C, Liu GY, He CH. Investigation on the Focal Area of Paragonimus in Dahongshan, Hubei Province. Acta Med Univ Sci Technol Huazhong. 1988,(04):285-87+316. Chinese.

119. Sun LQ,Li PG. Preliminary investigation of paragonimiasis in Yunyang area, Hubei Province. Chin J Parasitol Parasit Dis. 1986,(04):66. Chinese.

120. Zhang SK, Xue J, Yang SJ, Xiang XS, Huang WX. Hubei small bean snail and Chinese harlequin snail as the first intermediate host of Schistosoma lungworm found in western Hubei. J. Pathog. 1991,(01):27-28+84. Chinese.

121. Liu WX, Bei T, Chang XD, Tan TC, Zhu MS. First report on the investigation of paragonimiasis in Zhuxi County, Hubei Province. J Med P Ctl. 2002,(03):150-51. Chinese.

122. Tang LY, Li QJ, Zhang XJ, Zhang XX, Li DZ, Yang WQ. Epidemiological investigation of paragonimiasis in Hunan Province. J. Pathog. 1989,(04):271-75+318. Chinese.

123. Wang GR, Cao GH, Cao JS, Liang XS. Epidemiological investigation of paragonimiasis in Loudi area, Hunan Province. Chin J Parasitol Parasit Dis. 1991,(04):291-92. Chinese.

124. Wang GR, Yue YP, Cao JS, Chen WH. Epidemiological survey of paragonimiasis in Cangxi and Yangxi townships of Xinhua County, Hunan Province. J Clin Res. 1986,(05):315-16. Chinese.

125. Wang ZR. Preliminary study on species of paragonimus in Xinning County, hunan province. GX Med J. 1982,(04):194-96+2. Chinese.

126. Li YS, Lin CX, Zhou XM, Lin KQ, Cheng YZ, Li YR, et al. Discovery of mixed infection of low altitude Babesia and vesicular civet in Fuzhou, China. Chin J Zoonoses. 2003,(05):69-72. Chinese.

127. Zhao JL, Wang Y, Zhang L, Guo SR. Epidemiological investigation of paragonimiasis in Jincheng. Int J Med Parasit Dis. 2010,37(6):334-37. Chinese.

128. Lin CX, Huang BZ, Cheng YZ, Huang JM, Zhuo MY. Epidemiological investigation of *Paragonimus skrjabini* infection in Shunchang, Zhenghe and other areas of northern Fujian. Strait J Prevent Med. 2008,14(06):39. Chinese.

129. Li YR, He CR, Xie HG, Chen BJ, Zhang RY. Investigation on the new epidemic area of *Paragonimus skrjabini* in western Fujian. CA JST AJ CSTPCD. 2016,27(05):478-80. Chinese.

130. Wu NB, Pan GX, Long FD, Long XS. Investigation on the Epidemic Situation of Paragonimiasis in Qiandongnan Prefecture. Prac Prev Med. 1996,(04):208. Chinese.

131. Wang CQ, Wang DJ, Yang SJ, Li XF,LV QY. Ecological Study on the First Intermediate host of *Pagumogonimus skrjabini* in Xingshan County of the Three Gorges Reservoir Area. J Med P Ctl. 2009,25(08):572-74. Chinese.

131. Wang CQ, Yang SJ, Wang DJ, Pan HM, Lv QY, Qiu MS, et al. Study on the Pathogeny of *Pagumogonimus skrjabini* in Xingshan County of the Three Gorges Reservoir Area. J. Pathog. 2009,4(01):36-39. Chinese.

132. Zhu J, Zhu YX, Zhu MS, Wei RH. Investigation on natural foci of paragonimiasis in Shennongjia. Chin J Zoonoses. 2012,28(12):1255-57. Chinese.

132. Zhu J, Zhu YX, Zhu MS, Wei RH, Sang M. Etiology of *Pagumogonimus skrjabini* in Shennongjia. J Shanxi Univ Med. 2012,43(02):98-100. Chinese.

133. Li GM, Xu SA, Zhang XX, Li ZJ, Chen SZ, Wu QJ. Investigation on the prevalence of paragonimiasis caused by ecological imbalance. J Clin Res. 1990,(03):177-78. Chinese.

134. Kang ZB. A new snail host of *Paragonimus skrjabini*: Oncomelania archaea. Med J WH Univ. 1984,(04):354-57. Chinese.

135. Wang XS, Shi T, Wei J, Zhu MS. Studies on the Infection and Ecological Environment of Intermediate host of *Pagumogonimus skrjabini.* J Med P Ctl. 2006,(07):500-501. Chinese.

136. Gu XH, Liu WM. Epidemiological investigation of paragonimiasis in Mianyang area, Sichuan. B Dis Ctl Prev. 1987,(02):15-18. Chinese.

137. Yi DY, Luo XR, Liu JB. Epidemic investigation of paragonimiasis in the northern region of Sichuan Province. Chin J Zoonoses. 2008,(01):91-92. Chinese.

138. Luo XR, Yi DF, Liu JB, Zhou GX, Liu CH, Ceng MA, et al. Prevalence of paragonimiasis in Daba Mountains, Sichuan Province. Chin J Zoonoses. 1990,(04):60-61. Chinese.

139. Chen L, Lu D, Xu L. Zhong Bo. Epidemic status and trend analysis of paragonimiasis in Sichuan Province. Mod Prev Med. 2015,42(23):4230-33+4313. Chinese.

139. Chen L, Lu D, Xu L. Zhong Bo. Analysis of fixed monitoring results of paragonimiasis in Sichuan Province from 2011 to 2013. Chin J Schi Ctl. 2015,27(04):381-84+389. Chinese.

140. Cheng YL, Feng HP, Li YL, Liu ZH, Yi DY. Epidemic survey of paragonimiasis in the Fuling section of the Wujiang River Basin. Chin J Zoonoses. 1991,(06):59-60. Chinese.

141. Zhang XX, Duan JH, Bai XR, Tang LY, Li FJ. Investigation on the potential impact of Wuqiangxi water conservancy project on the prevalence of paragonimiasis. Chin J Zoonoses. 1995,(06):58-38. Chinese.

142. Li CF, Deng YY, Li JQ, Li ZR The first discovery of *Paragonimus skrjabini* in Baoshan area, Yunnan Province. J. Pathog. 2000,(04):66. Chinese.

143. Huang WX, Liu ZH, Zhang CH, Tian KQ. Epidemiological investigation report on paragonimiasis in Changyang County. China Public Health J. 1983,2(05):18. Chinese.

144. Zou YZ, Lv YM, Wang DB, Jiang YH, Ruan W, Xia SR, et al. Investigation on paragonimus infection in Quzhou city. Chin J Prev Med. 2008,(11):29. Chinese.

145. Ruan W, Yao LN, Yu KG, Xia SR, Chen HL. Epidemiological investigation of paragonimiasis in some areas of Zhejiang Province from 2000 to 2005. Prac Prev Med. 2006,(06):1488-89. Chinese.

146. Liu JF. Investigation of paragonimiasis in Yushan Village, Ninghai, Zhejiang in 2001. B Dis Ctl Prev. 2005,(01):97. Chinese.

147. Wu Y, Song FZ, Liu JF. Epidemiological investigation on Intermediate host of paragonimus in Ninghai County in 2004. B Dis Ctl Prev. 2006,(03):39-42. Chinese.

148. Yu YJ, Dai PY, Hu DB, Gu MX, Wang ZG, Wang B, et al. Investigation on the infection of paragonimus metacercaria in stream crabs in Ninghai County, Zhejiang Province, 2013-2015. Prac Prev Med. 2017,24(01):74-75. Chinese.

149. Chen SZ, Chen JB, Zheng P. Analysis on the infection rate of paragonimiasis in Yongjia County in 2013. Chin R H S admin. 2015,35(03):353-54. Chinese.

150. Fan PF, Chen DX. Preliminary report on infection of paragonimus metacercariae in southern Anhui Province. Curr Zool. 1973,(04):417. Chinese.

151. Fan PF, Chen DX, Zhang ZH, Liu JR. A Study on the Cyst and Adult of *Paragonimus westermani* in Southern Anhui Province. Curr Zool. 1979,(04):347-52. Chinese.

152. Zhang LS, Li TT, Zhang SQ, Huang JG, Zhai DJ, Yang RS. Investigation on Paragonimus infection in Xiuning County, Anhui Province. J Tro Dis Para. 2021,19(06):325-28. Chinese.

153. Liu DH, Guo JD, Zhang LS, Li XY, Zhu L,Jin w, et al. Investigation on paragonimus infection in Shitai County, anhui province. J Tro Dis Para. 2019,17(02):94-96. Chinese.

154. Wang BF, Xu AL. Investigation of Paragonimus metacercaria in Freshwater crab and morphological observation of metacercaria, metacercaria and adult worm. ZJ Med J. 1986,(02):63-64. Chinese.

155. Li DY, Tan QW, Quan CR. Investigation on the Natural Infection Type of *Paragonimus westermani* in a Crayfish. J Chin Med Univ. 1991,(03):190-91. Chinese.

156. Hong JL, Liu MD, Shao XY, Pan JZ. Investigation on Severe Cases and Epidemic Focus Caused by Diploid *Paragonimus westermani.* Chin J Zoonoses. 2012,28(04):403-404+409. Chinese.

157. Li YS, Cheng YZ, Chen QX, Tang ZG. Investigation on the etiology of paragonimiasis in Minqing County, Fujian Province. Wy Sci J. 1984,4(00):65-70. Chinese.

158. Mao QR. A PRELIMINARY INVESTIGATION ON EPIDEMIOLOGY OF PARAGONIASIS IN Shouning County, Fujian Province. Chin J Zoonoses. 1988,(01):29. Chinese.

159. Lin ZG, Li YS, Zhang ZP, Lin CX, Ding JZ, Cai CH, et al. Investigation on paragonimiasis in Yanping District, fujian province. J Tro Dis Para. 2003,(03):135-37+194. Chinese.

160. Chen HG, Zhou XM, Zhang SR, Peng WD, Wang WZ, Yuan JH, Cao XJ. Investigation on the Epidemic Status of Paragonimiasis in 5 Counties and 1 City in Northeast and South Jiangxi. Chin J Parasitol Parasit Dis. 1991,(S1):51-53. Chinese.

161. Lu YY, Shen HX, Kuang HC, Liu Q, Zhao TP, Long XS, et al. Preliminary Study on the Epidemic and Distribution of Paragonimus in the Northern Mountainous Areas of Guangzhou. Chin J Zoonoses. 2011,27(07):605-609. Chinese.

162. Zhao TP, Lu YY, Shen HX, Long XS, Wei HF, Luo JL. Investigation on snail species, crabs, and their infection with paragonimus in the northern mountainous areas of Guangzhou. J TROP MED. 2014,14(04):510-12. Chinese.

163. Li XW, Liu Q, Lu YY, Shen HX, Pan YZ, Liang HB Epidemic and Distribution of *Paragonimus westermani* in Zengcheng City, Northeast Suburb of Guangzhou Clin. Lab. Med. 2014(23):3243-44,3248. Chinese.

164. Wang J, Xu WM, Ruan W, Xia SR, Tang YM,Yang Y, et al. Investigation on the infection of metacercariae of paragonimus in stream crabs and population in Hangzhou City. Chin Prim Health Care. . 2009,23(05):68-69. Chinese.

165. Zhong HL, Xu ZB, Gao PZ. A preliminary investigation of paragonimiasis in Yichun and other regions of Heilongjiang Province and the discovery of subspecies of *Paragonimus westermani.* Curr Zool. 1979,(01):33-43. Chinese.

166. Shen JQ,Liu DY,Hu WQ. Report on the infection of the second Intermediate host of *Paragonimus westermani* in Liuyang County, Hunan Province. J GX Med Univ. 2001,(01):54-55. Chinese.

167. Fang YM, Hu XS, Ling ZL, Wang YB, Zheng RF, Tang YN Investigation on the infection of the second Intermediate host of paragonimus in Huangshan City. J Trop Dis Para. 2014,12(04):227-29. Chinese.

168. Chen BJ, Pei ZY, Li YS. Investigation on the Germplasm Resources and Infection of Paragonimus in Jian'ou City. J TROP MED. 2008,(02):150-51+178. Chinese.

169. Peng ZM, Cao HX, Wu HY. Report on the prevalence of paragonimiasis in the forest farm of Shangcun, Fenyi, Jiangxi Province. J Nc Univ(Med Sci). 1981,(03):76-79. Chinese.

170. Zhou XM, Wu ZD, Wiang WZ, Chen HG, Mao YS, Pan BR. Investigation Report on the Distribution of Human Parasites in Jingdezhen, Jiangxi Province. Chin J Parasitol Parasit Dis. 1994,(S1):216-18. Chinese.

171. Wang WZ, Pan BR, Zhou XM, Chen HG. The distribution status of paragonimiasis and paragonimiasis in Jiangxi Province. Chin J Parasitol Parasit Dis. 1994,(S1):135-39. Chinese.

172. Yan T,Li GL. Investigation on the infection of juvenile crabs in *paragonimus westermani* endemic areas in Jing'an County, jiangxi province. Chin J Parasitol Parasit Dis. 2002,(03):18. Chinese.

173. Xie ZM, Wu FD, Huang WC, Zhou XM. A preliminary report on the investigation of paragonimiasis in Sandu area of Tonggu County, Jiangxi Province. Jx Med J. 1980,(05):52-53. Chinese.

174. Lv YM, Wang DB, Ruan B, Yao LN, Xia SR, Chen HL, et al. Epidemiological investigation of paragonimiasis in Kaihua City. J. Pathog. 2008,(02):95+85. Chinese.

175. Huang YX, Zhang S, Shen Y, Liang J, Liu XW, Li YH, et al. Investigation on host infection of *Paragonimus westermani* in kuandian county. J Med P Ctl. 2016,32(04):420-21. Chinese.

176. Zhang YG, Dai D, Gong ZJ, Lin D, Yang ZQ. Analysis of the Epidemic Status of Paragonimus in Dandong District, Liaoning Province. J. Pathog. 2000,(02):52-54. Chinese.

177. Li DY, Zhou JS, Liu TC, Tan DK, Tan QW, Li XC, et al. Discovery and differences of two types of *Paragonimus westermani* in Fengcheng County, Liaoning Province J Chin Med Univ. 1990,(02):88-91+99. Chinese.

178. Li DY, Wang ER, Yu XH, Wang JC, Liu YZ, Jiang QA, Yang ER, et al. Epidemic situation of paragonimiasis in the Hunjiang River Basin of Kuandian County, Liaoning Province and exploration of seroepidemiological pathogen biology. J Chin Med Univ. 1985,(02):117-21+95. Chinese.

179. Wei ZR, Shen CK. Investigation on Intermediate host and pathogen of paragonimus in Longquan County. Chin J Zool. 1983,(04):5. Chinese.

180. Pan LX. Correction of mistakenly identified species of Sinopotamia sinensis in Meizhou City and investigation on infection of Sinopotamia sinensis in Pinghe with Paragonimus metacercaria. J TROP MED. 2002,(03):243-44. Chinese.

181. Cheng YZ,A. H. Toure, Zhang XH, Xu GF. Observation on the Infection and Ecological Habits of the Lung Fluke Cysticercariae in Hehuaxi Crab. Chin J Schi Ctl. 2001,(01):35-37. Chinese.

182. Huang LT. Analysis of paragonimus infection in stream crabs in Wuyang mountain area of Pingyang County. J Wz Med Univ. 1995,(S1):110. Chinese.

183. Zhao ZP, Ren ZX, Yan LY. Epidemiological Investigation Report on *Paragonimus westermani* in Shandong Province. J. Pathog. 1997,(01):80. Chinese.

184. Du YF, Hu SL. The first discovery of an endemic area of paragonimiasis in Shangyu County. Zhejiang M J. 1984,(01):60. Chinese.

185. Li DY, Han JS, Wang JC, Liu YZ, Yuan W, Wang XJ, et al. Investigation of paragonimiasis in Ji'an County, Tonghua. J Chin Med Univ. 1983,(01):47-49. Chinese.

186. Peng WD, Zhou XM, Dong CA, Wang WZ. The distribution pattern of *Paragonimus westermani* in the host population of crabs. Chin J Zoonoses. 1992,(05):24-26. Chinese.

187. Dong W,Peng WD. Paragonimiasis in Deshengguan, Mount Wuyi. J Nc Univ(Med Sci). 1981,(04):103-106. Chinese.

188. Chen JQ, Liu SH, Luo Y, Cai MS, Cheng YZ. Investigation on the Population of Freshwater crab and Paragonimus Infection in the Minjiang River Basin in the Middle Section of Mount Wuyi Mountain. Chin J Schi Ctl. 2021,33(06):590-99. Chinese.

189. Yin CC, Kang Y, Gong ZJ. Investigation on an outbreak of imported paragonimiasis. J. Pathog. 2001,(03):91. Chinese.

190. Han JS, Li DY, Yuan JH, Tan DW, Zhang DS. Recent Status and Observations on the Chromosomes of Adults: A Discussion on the Independence of the Ichun Subspecies of Schistosoma westermani. J Chin Med Univ. 1986,(01):6-10+75. Chinese.

191. Xu GQ, Xie Y, Lu HY, Zeng CL, Zhong LInvestigation on the infection of the second Intermediate host of paragonimus in Yichun. Jiangxi Sci. 1993,(04):250-51. Chinese.

192. Zhou TJ, Chen HQ, Hong JL. Dynamic Analysis of the Infection Rate of *Paragonimus westermani* in Freshwater Creek Crabs in Yongjia County Chin J Schi Ctl. 2013,25(06):655-56+671. Chinese.

193. Zhou QD, Jiang DS. Distribution of paragonimus in endemic areas of Yongjia County Chin J Prev Med. 1991,(02):23. Chinese.

194. Shi HH, Lu BJ, Zhao WJ, Hu XQ, Ma CL. Investigation on the infection of *Paragonimus westermani* in rural areas of Yuyao. Chin J Prev Med. 2008,(04):15. Chinese.

195. Ye XL, Ruan W, Liu SF, Mao SX, Yao SR, Yang TT. Investigation on the status of paragonimiasis foci in Songyang County, Zhejiang Province. J. Pathog. 2013,8(07):631-32+635. Chinese.

196. Yuan JX. Preliminary investigation report on the prevalence of paragonimiasis in Zhenhai County. Zhejiang Med J. 1984,(01):60-61. Chinese.

197. Zhu MS, Zhu J, Zhu YX, Wei RH, Yang SG. Epidemiological survey of prevalence of paragonimiasis in Danjiangkou reservior areas in Hubei Province. Chin Tro Med. 2011,11(11):1308-09. Chinese.

198. Yu L, Liu BY. Surveillance report on paragonimiasis in Pengzhou City, Sichuan Province, 2010-2012China Health Vis. 2013, 21(009):553-53. Chinese.

199. Liu ZY, Liu CF, Li BX, Tang DH, Liu M, Yuan XM, et al. Investigation on the prevalence of paragonimiasis in Kaijiang County in 2012. Prev Med Trib. 2013,19(04):255-57+260. Chinese.

200. Deng Y, Zhang YL, Gao LJ, Chen WQ, Zhang HW, Li P, et al. Investigation on Parasite Infection in Some Aquatic Products in Henan Province from 2013 to 2015. Mod Dis Ctl Prev. 2018,29(08):600-603. Chinese.

201. Hu XQ, Zhao Q, Liu ZY, Gui GY, Peng HW, Du MR. Analysis on the infection rate of paragonimiasis in Yongjia County in 2013. J Prev Med Info. 2015,31(02):136-39. Chinese.

202. Zhang J, Xia J, Zhang HX, Zhu H, Wu DN, Wan L, et al. Analysis of monitoring results of paragonimus infection in Hubei Province from 2018 to 2020. Chin J Parasitol Parasit Dis. 2021,39(05):600-605. Chinese.

203. Zeng DG. Investigation of paragonimiasis infection and Intermediate host in Chengkou County. J. Pathog. 1990,(04):276. Chinese.

204. Zhang GY, Guo EP, Yang SG. Distribution and Epidemic Trend of *Pagumogonimus skrjabini* in Shiyan City, Northwest Hubei Province. J TROP MED. 2009,9(02):206-207+187. Chinese.

205. Yang GA, Lu ZX, Qin JY. Investigation on the second Intermediate host of paragonimus in Tujia Miao Autonomous Prefecture, western Hubei Province, Freshwater crab and their carrying marsupials. Chin J Zool. 1990,(02):1-3. Chinese.

206. Yang GA. Investigation on the Natural reservoir of *paragonimus skrjabini* in Tujia Miao Autonomous Prefecture, western Hubei. Chin J Parasitol Parasit Dis. 1985,(01):65. Chinese.

206. Gu XH, Ren YQ. Investigation of paragonimiasis in Pingwu, Sichuan. Chin J Parasitol Parasit Dis. 1985,(01):65-66. Chinese.

207. Gu XH, Huang X. A NEW Intermediate host OF PULMONARY SCHISTOSOMA -- THE CHINESE CRAB (CRAB SINENSIS) FROM YANGTZE RIVER, SHANXIAN SUBSPECIES. Chin J P H. 1987,(03):159-60. Chinese.

208. Wei HW, Cheng YZ,Cao YP, Jiang DP, Lin CX. Investigation on new epidemic area of Paragonimus skrjabini in Zhenghe County, northern Fujian. J TROP MED. 2010,10(01):83-84+89. Chinese.

209. Lin GH, Zheng RD, Wu BC, Yan CL, Chen SH, Cheng YZ. Investigation on Freshwater crab in *Paragonimus skrjabini* foci in Hua'an County, fujian provinceInt J Med Parasit Dis. 2013,40(5):251-56. Chinese.

210. Xuan HJ, Wen J, Lu YY, Li XW, Shen HX, Huang X, et al. Epidemic status of paragonimiasis in Nankunshan Nature Reserve, Guangdong Province. J TROP MED. 2015,15(6):824-27. Chinese.

211. Li XW, Liu Q, Lu YY, Shao ZX, Deng JS, Liu CK, et al. Investigation on the Natural Focus of Paragonimus in Xinfeng County, Northern Suburb of Guangzhou City. J MED RES. 2013,42(09):74-76. Chinese.

212. Li ZL, Niu H, Zhang XL, Wang Y, Chen WB, Qian BZ, et al. Epidemiological investigation of paragonimiasis in Hejiang County and its adjacent areas: with 2 cases of liver damage caused by paragonimiasis. Para Infect Dis. 2009,7(02):66-70. Chinese.

213. Cui J, Wang ZQ, Lin XM, Mao FR, Jin XX, Wu F, et al. Epidemiological investigation of paragonimiasis in Nanyang area, Henan Province. J. Pathog. 1999,(01):90. Chinese.

214. Chen GX, Chang J, Zhang XQ, Li QH. A PRELIMINARY INVESTIGATION ON Freshwater crab, THE SECOND Intermediate host OF PARAGONIASIS IN HENAN PROVINCE. Chin J Zool. 1982,(04):1-3+65. Chinese.

215. Zhong HL. Investigation on paragonimiasis in Wuli commune, Hefeng County, Hubei province. J N Med. 1978,(01):18-20+10. Chinese.

216. Xu ZM, Li L, Wu XY, Zhao HF, Du AP, Hu S, et al. Host Infection of *Paragonimus skrjabini* and Questionnaire Survey in a Natural Village in Hubei Province. Chin J Parasitol Parasit Dis. 2008,(01):75-76. Chinese.

217. Tang C, Jiang CF, Li DR. Discovery of paragonimiasis in Shennongjia, hubei province. Acta Med Univ Sci Technol Huazhong. 1986,(05):374. Chinese.

218. Lin H, Zhang SQ, Yang LT. Regional distribution of the second Intermediate host of paragonimus and its metacercaria carrying status in Hubei Province. J Public Health Prev. 1991,(04):20-22+13. Chinese.

219. Shen H, Peng DH, Li Y, Yu XT, Luo GR, Peng ZQ. Preliminary report on the investigation of paragonimiasis and its pathogens in Enshi County, Hubei Province. Med J WH Univ. 1985,(03):273-78. Chinese.

220. Dong XR, Cui XF, Huang GQ. Prevalence of paragonimiasis in Xingshan County, Hubei Province. CA JST AJ CSTPCD. 2013,24(05):101-102. Chinese.

221. Sun LQ, Li PG. *Paragonimus skrjabini* epidemic area found in Zhuxi, Hubei. Chin J Parasitol Parasit Dis. 1985,(01):67. Chinese.

222. Li H, Lin XM, Han QX, Chang J, Xu BL. Investigation on the epidemic situation of paragonimiasis in Lushi County. Mod Dis Ctl Prev. 2000,(02):108. Chinese.

223. Zhu MS, Zhu YX, Song MH, Wang SJ. Epidemiological investigation of *paragonimiasis skrjabini* in the reservoir area of the South–North Water Transfer Project. J Med P Ctl. 2006,(07):505. Chinese.

224. Li YS, Cheng YZ, Lin CX, Lu CJ, Ye XP, Wu JY, et al. Discoveries of Pagumogonimus vesiculatum in Fujian Province. Chin J Parasitol Parasit Dis. 2000,(05):42-46. Chinese.

225. Yang YX, Yu XG, Luo ZS, Peng LB, Yun LJ. Seroepidemiological investigation of paragonimiasis in the head area of the second phase water storage reservoir of the Three Gorges Project. Chin Tro Med. 2011,11(05):548-50. Chinese.

226. Zhang XL, Wang Y, Huang Y, Chen GG, Huang ZG, Huang Y, et al. The impact of environmental changes in the Three Gorges reservoir area after the construction of the dam on the prevalence of paragonimiasis. J. Pathog. 2002,(01):34-36. Chinese.

227. Zhang XL, Wang Y, Zhang JR, Yang S. A Study on the Epidemic Status and Clinical Manifestations of Pulmonary Clonorchiasis in the Three Gorges Reservoir Area of Chongqing: An Analysis of the Causes of Misdiagnosis of 58 Cases of *Paragonimus skrjabini*. NA. 83. 5875. Chinese.

228. Xue JD, Hou YS, Han ZX, Kong XJ. Epidemiological investigation report on paragonimiasis in Tongguan County, Shaanxi Province Shannxi M J. 1980,(10):32-34+65. Chinese.

229. Zhang GY, Yang SG, Guo EP. Analysis on the blocking factors of epidemic of *Paragonimus skrjabini* in Shiyan urban area. Chin Tro Med. 2012,12(02):213-14. Chinese.

230. Su TC, Yang MX, Zhang PX, Zhou SL. Discoveries of *Paragonimus skrjabini* in Fang County, Hubei Province. J. Pathog. 1983,(01):63. Chinese.

231. Yi DY, Luo XR, Liu JB. Epidemiological investigation of paragonimiasis in Yibin area, Sichuan Province. J. Pathog. 1991,(04):309. Chinese.

232. Zhang JR, Wang Y, Yang S, Zhang XL. Prevalence of paragonimiasis in Wushan and Fengjie County and misdiagnosis analysis of 46 cases. Chin J Zoonoses. 2004,(02):163-64. Chinese.

233. Wang CY, Hai NK. Investigation on a case of paragonimiasis caused by eating raw crabs. J. Pathog. 1989,(04):311-12. Chinese.

234. Ba WF, He XY, Huang ZM. Investigation on the infection of Freshwater crab, the second Intermediate host of paragonimus in Yuxi area. Chin J Zool. 1996,(02):22-23. Chinese.

235. Zhang WQ, Guo YM, Li W, Zhou BJ, Wang WL. Investigation on the second Intermediate host of paragonimus in yunnan province. J Kunming Med Univ. 2015,36(12):17-20. Chinese.

236. Wang GX, Huang YQ, Chen WB, Shen JB, Wang M, Mao YY, et al. Follow up investigation of 24 cases of paragonimiasis in Weixin County, Yunnan Province Sichuang. J Zool. 2012,31(04):605-607. Chinese.

237. Dai TT, Zhou BJ, Shi SL, Li CY, Zhang WQ, Wang H. Investigation of Paragonimus Species and the Second Intermediate host in the Midwest of Yunnan Province. J. Pathog. 2013,8(06):544-46. Chinese.

238. Yi DY, Luo XR. Natural infection of paragonimus japonicus found in two Freshwater crab in sichuan province. Curr Zool. 1992,(04):442. Chinese.

239. Zhang XL, Wang Y, Wang GX, Chen WB, Xie H, Niu H, et al. Distribution and clinical features of *Paragonimiasis skrjabini* in Three Gorges Reservoir Region. Parasitol Int. 2012,61(4):645-49. English.

240. Zhang GY, Guo EP, Yang SG. The Impact of Heightening the Danjiangkou Dam on the Epidemic of *Paragonimus skrjabini* in the Middle and Upper Reaches of the Han River. Mod Prev Med. 2010,37(02):372-73+391. Chinese.

241. Zhang R. Title: Comprehensive report on epidemiological investigation of paragonimiasis in various regions (subtitle: investigation of *paragonimiasis skrjabini* in Nanzhang County, Hubei Province)Chin J Parasitol Parasit Dis. 1991, (3):79-80. Chinese.

242. Zhang LS, Li TT, Zhang SQ, Huang JG, Zhai DJ, Yang RS. Investigation on Paragonimus infection in Xiuning County, Anhui Province. J Tro Dis Para. 2021,19(06):325-28. Chinese.

243. Zhu MS, Qian BZ, Shan SG. Detection of Paragonimiasis by dot immunogold filtration asser（DIGFA-kit）and investigation of epidemiology. J S Univ Med. 2008,39(12):1090-91. Chinese.
