# Supplementary figures and images for: Infection and biogeographical characteristics of Paragonimus westermani and P. skrjabini in humans and animal hosts in China: A systematic review and meta-analysis
Source: PLoS Negl Trop Dis. 2024 Aug 5;18(8):e0012366. doi: 10.1371/journal.pntd.0012366 (PMC11326572; doi:10.1371/journal.pntd.0012366)

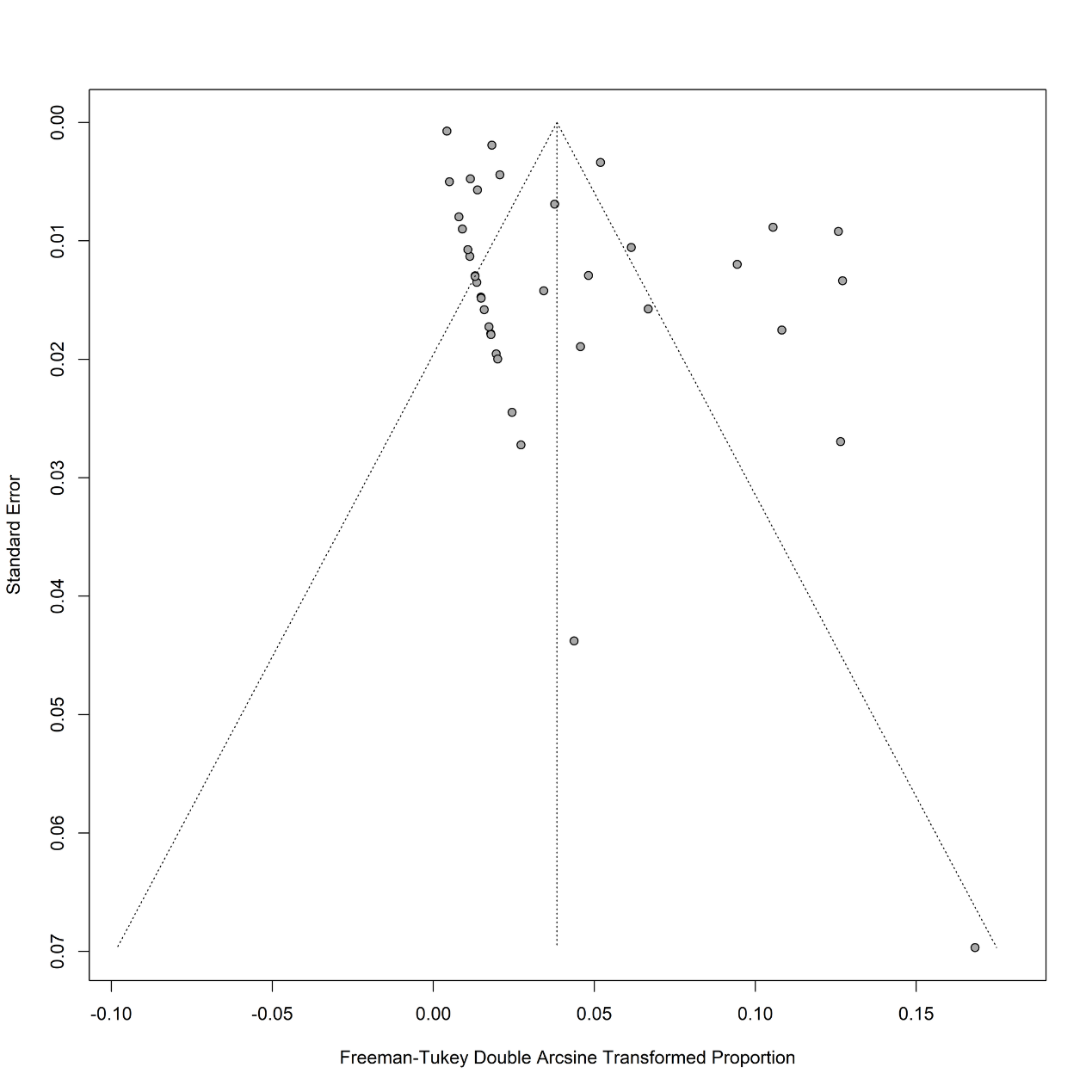


S2a_Fig


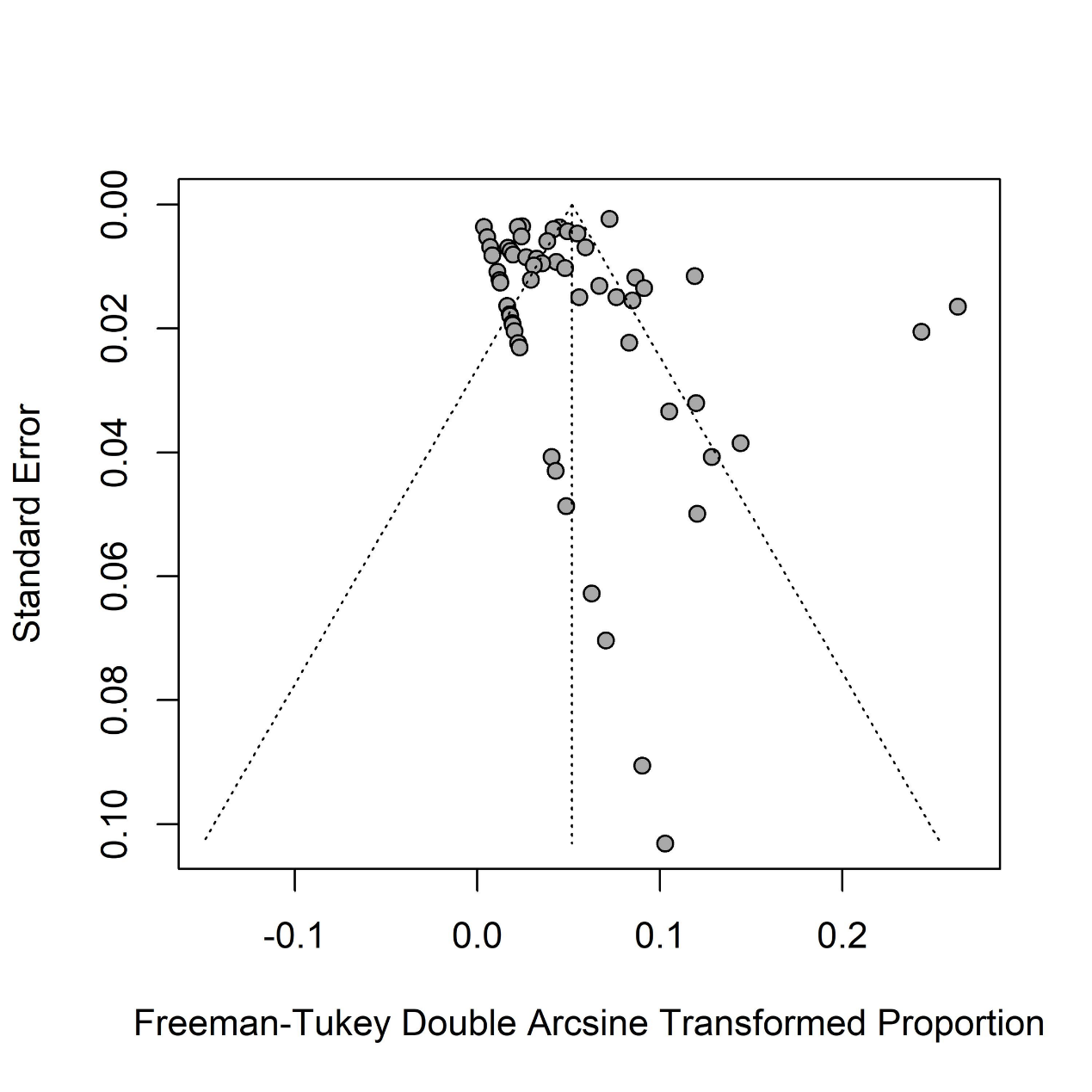


S2b_Fig


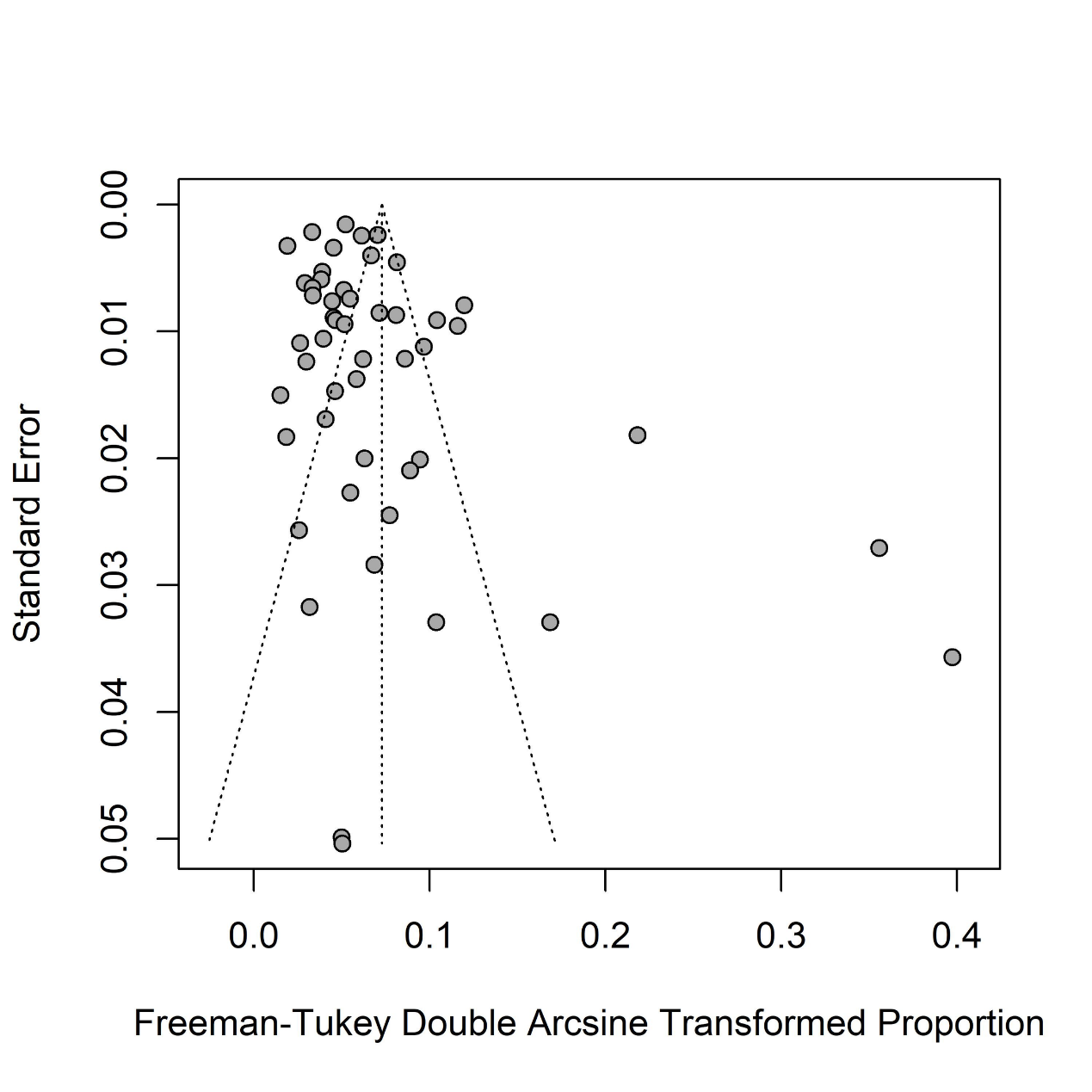


S2c_Fig


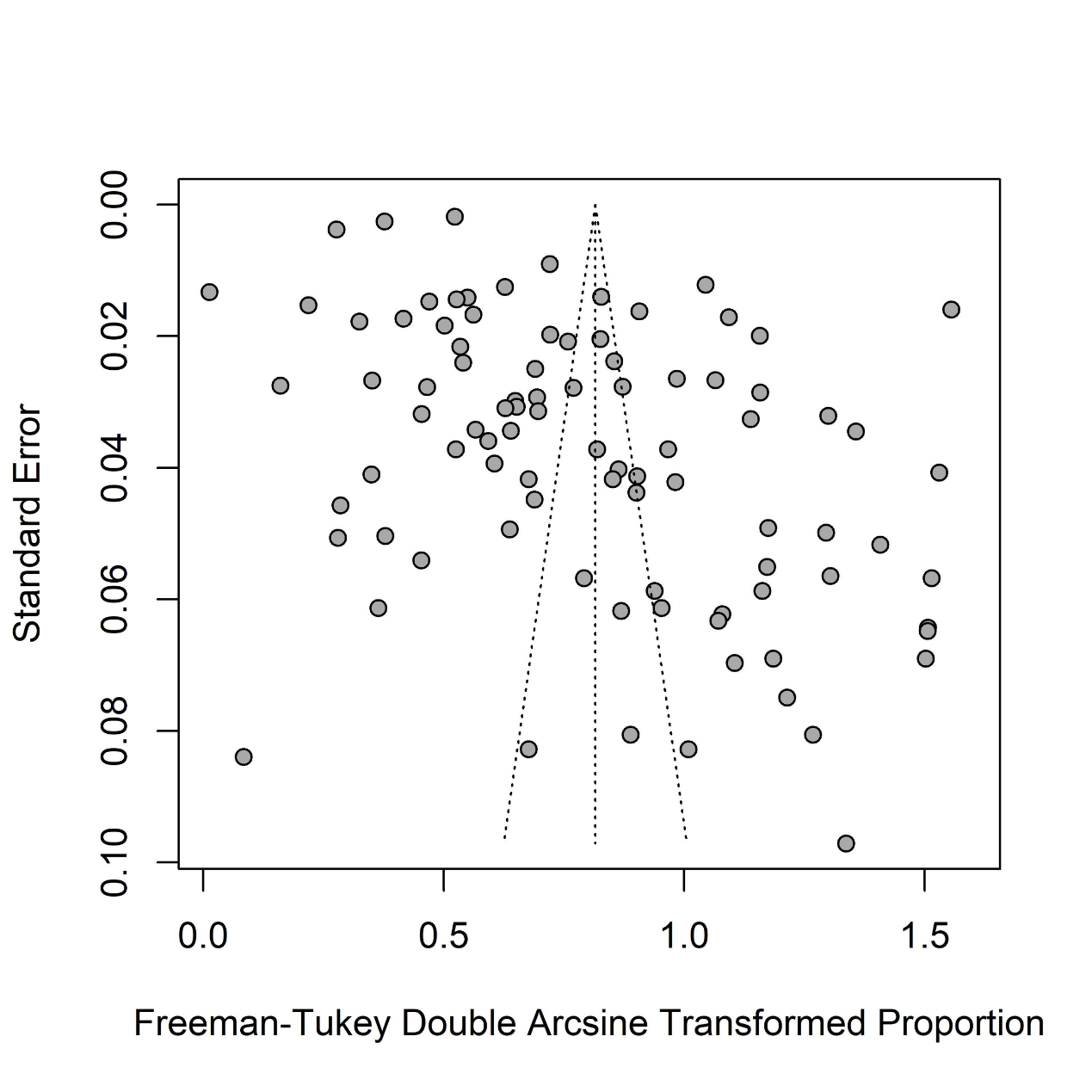


S2d_Fig


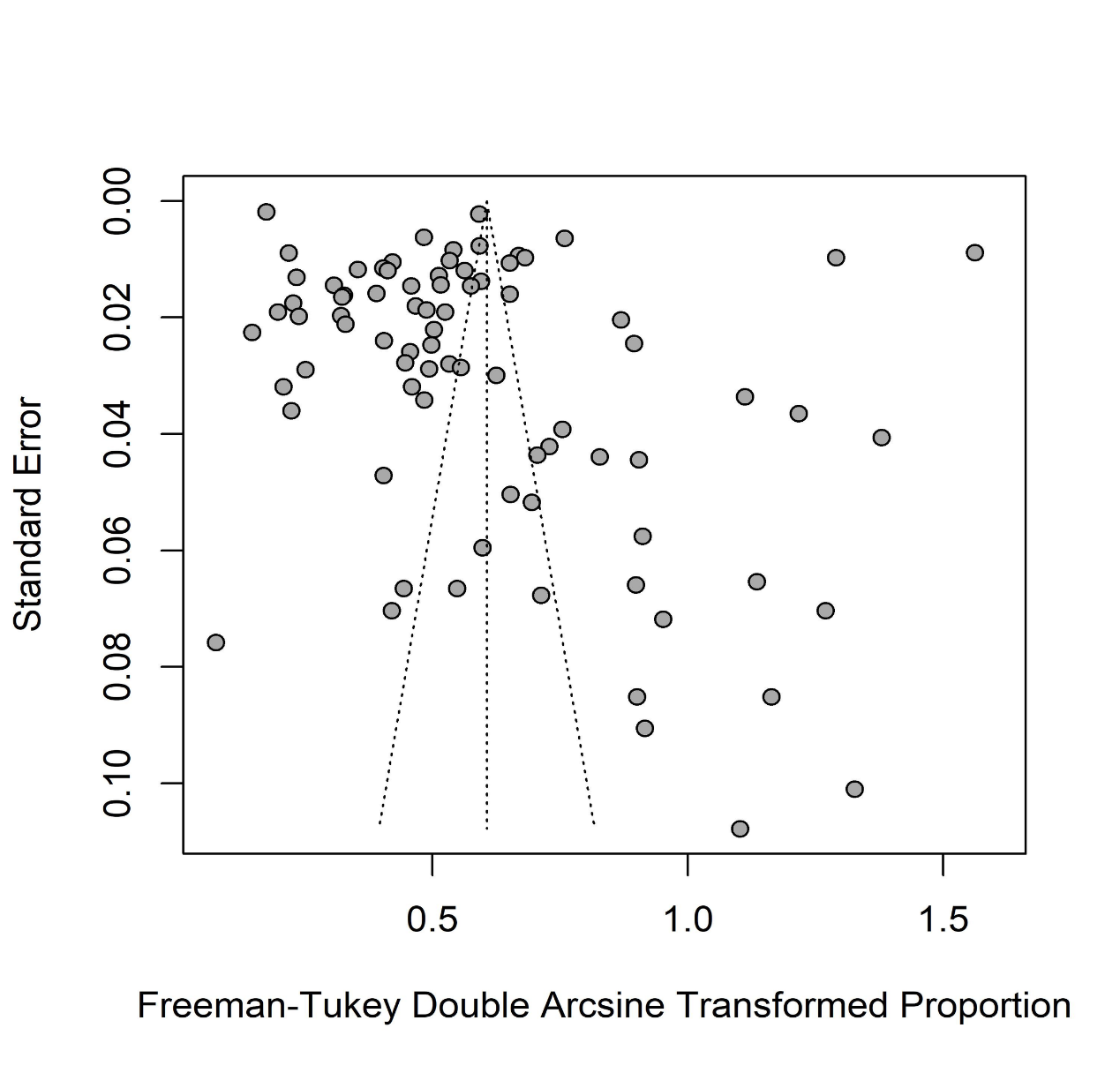


S2e_Fig


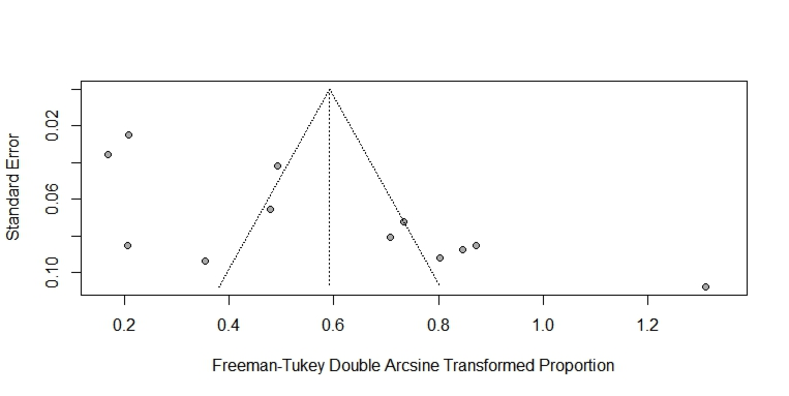


S2f_Fig


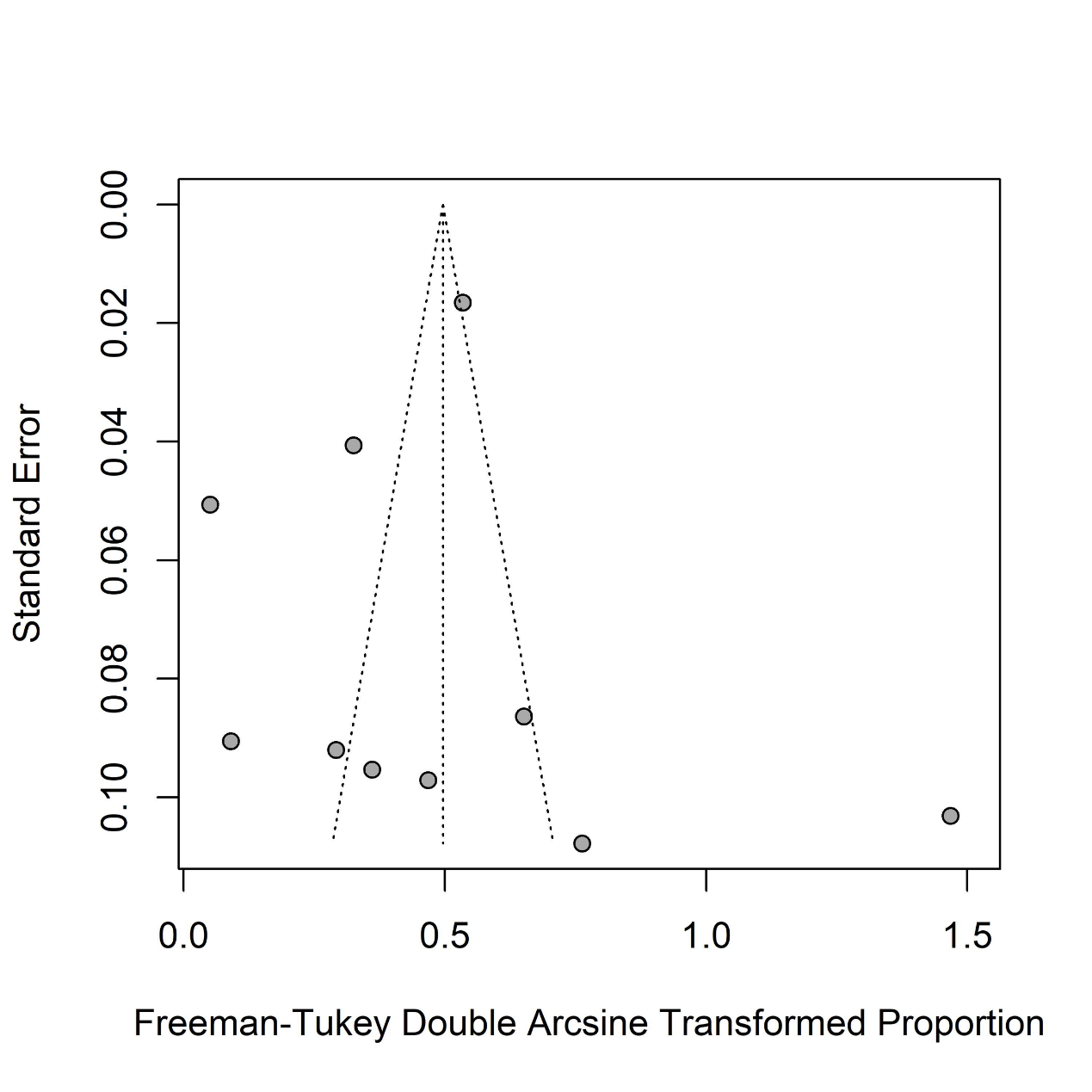


S2g_Fig

Supplement: S2 Fig — Funnel plot for assessing publication bias in studies reporting prevalence of Paragonimus species in humans, the first intermediate host, the second intermediate host, and animal reservoirs (a) Paragonimus in humans; (b) P. skrjabini in the first intermediate host; (c) P. westermani in the first intermediate host; (d) P. skrjabini in the second intermediate host; (e) P. westermani in the second intermediate host; (f) P. skrjabini in animal reservoir; (g) P. westermani in animal reservoir. (DOC) [file pntd.0012366.s013.doc]
