# Supplementary material for: Infection and biogeographical characteristics of Paragonimus westermani and P. skrjabini in humans and animal hosts in China: A systematic review and meta-analysis
Source: PLoS Negl Trop Dis. 2024 Aug 5;18(8):e0012366. doi: 10.1371/journal.pntd.0012366 (PMC11326572; doi:10.1371/journal.pntd.0012366)
Supplement: Attachment — Submitted filename: Revision Paragonimus.pdf [file pntd.0012366.s014.pdf]

PLOS Neglected Tropical Diseases

Infection and biogeographical characteristics of Paragonimus westermani and P. skrjabini in humans and animal hosts in China: a systematic review and meta-analysis

--Manuscript Draft--

**Manuscript Number:**	PNTD-D-24-00165
**Full Title:**	Infection and biogeographical characteristics of Paragonimus westermani and P. skrjabini in humans and animal hosts in China: a systematic review and meta-analysis
**Short Title:**	Infection and biogeographical characteristics of Paragonimus in China
**Article Type:**	Research Article
**Keywords:**	Paragonimus; prevalence; meta-analysis; temperature; precipitation; animal hosts; China
**Abstract:**	Abstract<br>Background<br>Paragonimiasis, primarily caused by Paragonimus westermani and P. skrjabini in China, is a common food-borne parasitic zoonosis. Despite numerous epidemiological surveys conducted over the past decades, the national distribution of Paragonimus infection and its associated environmental determinants remain poorly understood. In this paper, we summarize the infection of P. westermani and P. skrjabini and describe key biogeographical characteristics of the endemic areas in China.<br>Methods<br>Data on Paragonimus infection in humans, snails, second intermediate hosts, and animal reservoirs were extracted from eight electronic databases. A random-effects meta-analysis model was used to estimate the pooled prevalence. All survey locations were georeferenced and plotted on China map, and scatter plots were used to illustrate the biogeographical characteristics of regions reporting Paragonimus infection.<br>Results<br>A total of 28,948 cases of human paragonimiasis have been documented, with 2,401 cases reported after 2010. Among the 11,443 cases with reported ages, 88.05% were children or adolescents. The pooled prevalence of P. skrjabini is 0.45% (95% CI: 0.27 – 0.66%) in snails, 31.10% (95% CI: 24.77 – 37.80%) in the second intermediate host, and 20.31% (95% CI: 9.69 – 33.38%) in animal reservoirs. For P. westermani, the pooled prevalence is 0.06% (95% CI: 0.01 – 0.13%) in snails, 52.07% (95% CI: 43.56 – 60.52%) in the second intermediate host, and 21.40% (95% CI: 7.82 – 38.99%) in animal reservoirs. P. westermani and P. skrjabini are primarily distributed in regions with low altitude, high temperature, and high precipitation. In northeastern China, only P. westermani infections have been documented, with no presence of P. skrjabini, while in more southern areas, infections of both P. westermani and P. skrjabini have been reported.<br>Conclusions<br>Paragonimiasis remains prevalent in China, particularly among children and adolescents. Variations exist in the intermediate hosts and geographical distribution of P. westermani and P. skrjabini. Additionally, temperature and precipitation may influence the distribution of Paragonimus.
**Additional Information:**	
**Question**	**Response**
**Government Employee**<br><br>Are you or any of the contributing authors an employee of the United States government?<br><br>Manuscripts authored by one or more US	No - No authors are employees of the U.S. government.

Powered by Editorial Manager® and ProduXion Manager® from Aries Systems Corporation

Government employees are not copyrighted, but are licensed under a CC0 Public Domain Dedication, which allows unlimited distribution and reuse of the article for any lawful purpose. This is a legal requirement for US Government employees.<br><br>This will be typeset if the manuscript is accepted for publication.	
**Financial Disclosure**<br><br>Enter a financial disclosure statement that describes the sources of funding for the work included in this submission. Review the submission guidelines for detailed requirements. View published research articles from *PLOS Neglected Tropical Diseases* for specific examples.<br><br>This statement is required for submission and **will appear in the published article** if the submission is accepted. Please make sure it is accurate.<br><br>**Funded studies**<br>Enter a statement with the following details:<br>• Initials of the authors who received each award<br>• Grant numbers awarded to each author<br>• The full name of each funder<br>• URL of each funder website<br>• Did the sponsors or funders play any role in the study design, data collection and analysis, decision to publish, or preparation of the manuscript?<br><br>Did you receive funding for this work?	Yes
Please add funding details.<br>as follow-up to "**Financial Disclosure**<br><br>Enter a financial disclosure statement that describes the sources of funding for the work included in this submission. Review the submission guidelines for detailed requirements. View published research articles from *PLOS Neglected Tropical*	This research was funded by the Shandong Provincial Natural Science Foundation (https://cloud.kjt.shandong.gov.cn/) (grant no. ZR2019MH093, ZR2023MH313), the Shandong Provincial Youth Innovation Team Development Plan of Colleges and Universities (http://edu.shandong.gov.cn/) (grant no. 2019-6-156, Lu-Jiao), the National Parasite Resource Bank (https://www.most.gov.cn/index.html) (grant no. NPRC-2019-194-30), the Quality Education Teaching Resources Project of Shandong Province and Weifang Medical University (http://edu.shandong.gov.cn/) (grant no. SDYAL2022152, 22YZSALK01, 23YJSALK01), Joint Research Program of China National Center for Food Safety Risk Assessment (https://cfsa.net.cn/) (grant no. LH2022GG08,

Diseases for specific examples.<br><br>This statement is required for submission and **will appear in the published article** if the submission is accepted. Please make sure it is accurate.<br><br>**Funded studies**<br>Enter a statement with the following details:<br>• Initials of the authors who received each award<br>• Grant numbers awarded to each author<br>• The full name of each funder<br>• URL of each funder website<br>• Did the sponsors or funders play any role in the study design, data collection and analysis, decision to publish, or preparation of the manuscript?<br><br>Did you receive funding for this work?"	LH2022GG02), and the National Natural Science Foundation of China (https://www.nsfc.gov.cn/) (grant no. 81902905).
Please select the country of your main research funder (please select carefully as in some cases this is used in fee calculation).<br>as follow-up to "**Financial Disclosure**<br><br>Enter a financial disclosure statement that describes the sources of funding for the work included in this submission. Review the submission guidelines for detailed requirements. View published research articles from *PLOS Neglected Tropical Diseases* for specific examples.<br><br>This statement is required for submission and **will appear in the published article** if the submission is accepted. Please make sure it is accurate.	CHINA - CN

Powered by Editorial Manager® and ProduXion Manager® from Aries Systems Corporation

**Funded studies**<br>Enter a statement with the following details:<br>• Initials of the authors who received each award<br>• Grant numbers awarded to each author<br>• The full name of each funder<br>• URL of each funder website<br>• Did the sponsors or funders play any role in the study design, data collection and analysis, decision to publish, or preparation of the manuscript?<br><br>Did you receive funding for this work?"	
**Competing Interests**<br><br>On behalf of all authors, disclose any competing interests that could be perceived to bias this work.<br><br>This statement will be typeset if the manuscript is accepted for publication.<br><br>Review the instructions link below and PLOS NTDs' competing interests policy to determine what information must be disclosed at submission.	The authors have declared that no competing interests exist.
**Data Availability**<br><br>Provide a **Data Availability Statement** in the box below. This statement should detail where the data used in this submission can be accessed. This statement will be typeset if the manuscript is accepted for publication.<br><br>Before publication, authors are required to make all data underlying their findings fully available, without restriction. Review our PLOS Data Policy page for detailed information on this policy. Instructions for writing your Data Availability statement can be accessed via the Instructions link below.	All relevant data are within the manuscript and its Supporting Information files.

Infection and biogeographical characteristics of *Paragonimus westermani* and *P. skrjabini* in humans and animal hosts in China: a systematic review and meta-analysis

Kai Liu[1#], Yuan-Chao Sun[1#], Rui-Tai Pan[1], Ao-Long Xu[1], Han Xue[1], Na Tian[1], Jin-Xin Zheng[2], Fu-Yan Shi[1*], Yan Lu[2*], Lan-Hua Li[1*]

[1]School of Public Health, Shandong Second Medical University, Weifang, Shandong 261053, PR China.

[2]National Institute of Parasitic Diseases, Chinese Center for Disease Control and Prevention, Shanghai, China; Key Laboratory of Parasite and Vector Biology, Ministry of Health, WHO Collaborating Center for Malaria, Schistosomiasis and Filariasis, Shanghai, China

*Corresponding author

E-mail: orchid8@sina.com (LH-L); lyyaner@163.com (YL); shifuyan@126.com (FY-S).

[#]These authors contributed equally to this work, and shared first authorship is acknowledged between Kai Liu and Yuan-Chao Sun.

All authors read and approved the final manuscript.

// Abstract

Background

Paragonimiasis, primarily caused by *Paragonimus westermani* and *P. skrjabini* in China, is a common food-borne parasitic zoonosis. Despite numerous epidemiological surveys conducted over the past decades, the national distribution of *Paragonimus* infection and its associated environmental determinants remain poorly understood. In this paper, we summarize the infection of *P. westermani* and *P. skrjabini* and describe key biogeographical characteristics of the endemic areas in China.

Methods

Data on *Paragonimus* infection in humans, snails, second intermediate hosts, and animal reservoirs were extracted from eight electronic databases. A random-effects meta-analysis model was used to estimate the pooled prevalence. All survey locations were georeferenced and plotted on China map, and scatter plots were used to illustrate the biogeographical characteristics of regions reporting *Paragonimus* infection.

Results

A total of 28,948 cases of human paragonimiasis have been documented, with 2,401 cases reported after 2010. Among the 11,443 cases with reported ages, 88.05% were children or adolescents. The pooled prevalence of *P. skrjabini* is 0.45% (95% *CI*: 0.27 – 0.66%) in snails, 31.10% (95% *CI*: 24.77 – 37.80%) in the second intermediate host, and 20.31% (95% *CI*: 9.69 – 33.38%) in animal reservoirs. For *P. westermani*, the pooled prevalence is 0.06% (95% *CI*: 0.01 – 0.13%) in snails, 52.07% (95% *CI*: 43.56 – 60.52%) in the second intermediate host, and 21.40% (95% *CI*: 7.82 – 38.99%) in

animal reservoirs. *P. westermani* and *P. skrjabini* are primarily distributed in regions with low altitude, high temperature, and high precipitation. In northeastern China, only *P. westermani* infections have been documented, with no presence of *P. skrjabini*, while in more southern areas, infections of both *P. westermani* and *P. skrjabini* have been reported.

Conclusions

Paragonimiasis remains prevalent in China, particularly among children and adolescents. Variations exist in the intermediate hosts and geographical distribution of *P. westermani* and *P. skrjabini*. Additionally, temperature and precipitation may influence the distribution of *Paragonimus*.

Author summary

Paragonimiasis, a foodborne zoonotic parasitic disease caused by lung flukes (*Paragonimus*), remains a significant neglected public health threat in many Asian countries, including China. Human infection occurs through the ingestion of raw or undercooked freshwater crab or crayfish containing the metacercariae stage. Given the popularity of consuming raw or undercooked freshwater products in many areas of China, understanding the infection status and spatial distribution of *Paragonimus* in humans and animal hosts is crucial for controlling paragonimiasis. Our study provides a comprehensive summary of the infection levels of the two most important zoonotic *Paragonimus* species, *P. westermani* and *P. skrjabini*, in humans and animal hosts in China, along with a description of the spatial distribution and environmental characteristics of their endemic areas. We observe a wide distribution of *Paragonimus* infection in China, with a significant infection rate found in freshwater crabs and

crayfish. Our findings underscore the importance of avoiding the consumption of raw or undercooked freshwater products to prevent foodborne diseases, including paragonimiasis.

Introduction

Paragonimiasis is a food-borne zoonotic disease caused by several species of lung flukes belonging to genus *Paragonimus* [1]. It typically causes subacute to chronic pneumonia. The symptoms, including chronic cough, chest pain, dyspnea, and hemoptysis, mimic those of tuberculosis and lung cancer [2]. Human paragonimiasis is widely distributed in Asia, Americas, and Africa, and is still a significant neglected public health threat in China. An estimated 293.8 million individuals are at risk of *Paragonimus* infection, with 195 million of them residing in China [3, 4].

More than 30 *Paragonimus* species have been documented in China, among which *P. westerman* and *P. skrjabini* are the most important zoonotic species [2, 5]. *P. westermani* (Japanese lung fluke or oriental lung fluke) is most commonly distributed in eastern Asia and in South America, and is the most common cause of human paragonimiasis. *P. skrjabini* is especially prevalent in China, with cases appearing more recently in India and Vietnam as well. *P. westermani* followed by *P. skrjabini* are the major pathogens for human paragonimiasis in China.

Parasites of *Paragonimus* spp. have a three-host life cycle, with aquatic snails serving as the first intermediate host, freshwater decapod crustaceans as the second intermediate host, while human and other mammals as the definitive host. Human infection is acquired by eating inadequately cooked or pickled freshwater crabs or

cray fishes containing the infective forms called metacercariae [6, 7]. Drinking untreated stream or river water is also considered to be a possible route of infection [8].

Given the three host nature of the parasite and the fact that consuming raw or undercooked freshwater products is still popular in many areas of China, the infection status of *Paragonimus* in animal hosts is closely related to the epidemic of human paragonimiasis [9]. Therefore, comprehending the level of infection in animals will provide valuable insights for controlling human paragonimiasis. However, prevalence estimates of *Paragonimus* infection in the literature vary greatly across different studies. To date, there has been no comprehensive estimation of *Paragonimus* infection in humans and animal hosts. In addition, very few attempts at the spatial and environmental characteristics of *Paragonimus* infection in China have been made. Consequently, the aims of the current study are to summarize the infection level of two most important zoonotic *Paragonimus* species, *P. westermani and P. skrjabini*, in humans and animal hosts in China, and to describe the spatial distribution and environmental characteristics of their endemic areas.

Method

Literature retrieval and selection

This systematic review followed the Preferred Reporting Items for Systematic Reviews and Meta-analyses (PRISMA) reporting guidelines [10].

A systematic literature search was conducted to identify all studies reporting *Paragonimus* infection in humans and animals from inception to January 1, 2024, using the following electronic databases: China National Knowledge Infrastructure

(CNKI), Chinese Wanfang database (CWFD), Chongqing VIP, SinoMed, Medline, Embase, PubMed, and Web of Science. Full-text search was performed using the terms ‘paragonimiasis’, ‘*Paragonimus*’, ‘lung fluke’, ‘lung trematode’, in conjunction with ‘China’. The search was limited to English and Chinese languages.

After removing duplicates, two reviewers (KL and YC-S) independently reviewed all the titles and abstracts, with assistance of a third reviewer (RT-P) to reach a consensus in case of disagreement. Subsequently, the full texts were assessed for inclusion by the same reviewers. All studies included in the meta-analysis were published in English or Chinese, and were primary research articles, and epidemiological studies reporting infection rate of *Paragonimus* in humans and animal hosts. Studies were further excluded from meta-analysis if they were letters to the editor, non-epidemiological studies, or had a sample size of fewer than 20 [11]. Additionally, we collected case reports and case series of human infections to summarize the characteristics of cases of human paragonimiasis.

Data extraction and quality assessment

The following information was extracted from the included articles: title, first author, language, year of publication, year of investigation, study location, *Paragonimus* species, diagnostic techniques used in the study, sample size, number of positive cases, infection rate, and taxonomic category of animal host for infection in animals. In population-based surveys, the participants first underwent immunological testing (usually skin testing), and those who tested positive further underwent etiological testing. In this case, the infection rate was calculated using the total number of

participants as the denominator, with etiologically confirmed positives as the numerator.

Two reviewers (KL and YC-S) independently evaluated the quality of each included study using a standardized assessment tool developed by Hoy [12]. This tool provides ten items to access the risk of bias, with each item given a score of 0 or 1 for the absence or presence of bias. A summary score of 0–3 indicates a low risk of bias, 4–6 indicates a moderate risk of bias, and 7–10 indicates a high risk of bias.

Statistical analysis

Freeman-Tukey double arcsine transformation was used to normalize the infection rate and ensure the validity of subsequent analyses [13]. Heterogeneity across studies was assessed using Cochran's Q test and I^2 statistics, where I^2 statistics quantified the percentage of variation across studies (with I^2 values indicating low, moderate, and high heterogeneity at 25%, 50%, and 75%, respectively). If the heterogeneity is statistically significant, a random-effects model was used for meta- analysis; otherwise, a fixed-effects model was used [14, 15]. The random-effects model was ultimately used to estimate the pooled prevalence in this study, following the results of the heterogeneity test. Additionally, subgroup and meta- regression analyses were employed to explore the potential source of heterogeneity across studies and assess the effects of moderators on the infection rates.

R^2, QM and QE statistics were utilized to interpret the results of subgroup and meta-regression analyses [11]. R^2 represents the proportion of true heterogeneity that can be explained by the moderator; QM and its *P*-value determine the significance of the moderators in explaining heterogeneity; and QE and its *P*-value evaluate the significance of unexplained residual heterogeneity [16, 17].

Funnel plots and Egger's test were employed to assess potential publication bias. Sensitivity analyses were conducted to evaluate the robustness of the pooled estimate [18, 19]. Initially, outlier analyses were performed using Baujat plots. Studies located in the top right quadrant of the Baujat plot, or with studentized residuals exceeding 2 in absolute value, were considered potential outliers. After removing identified outliers, the overall pooled prevalence estimates were recalculated and compared with the main findings. Furthermore, we examined whether excluding smaller-sample data points (i.e., data points with the lowest quintile of sample sizes) yielded findings similar to the main results.

All statistical analyses were performed using R4.2.1 software (Lucent Technologies, Jasmine Mountain, USA). For all tests, p values less than 0.05 were considered statistically significant.

Data collection on environmental factors and visualization of the spatial distribution and biogeographical characteristics

Baidu Map was used to determine the latitude and longitude coordinates of each study location. For human infection, all etiological confirmed paragonimiasis cases documented in population surveys, case reports, and case series were included in the spatial analyses. Environmental factors for each location, including annual mean temperature, annual precipitation, mean temperature of warmest quarter, precipitation of warmest quarter, mean temperature of coldest quarter, precipitation of coldest quarter were obtained from the WorldClim database (https://www.worldclim.org/) [20]. Altitude data was obtained from the Space Shuttle Radar Topography Mission (SRTM, http://www.gscloud.cn/) [21].

To visualize the spatial distribution of *P. westermani* and *P. skrjabini* infection, we georeferenced the etiologically definite human paragonimiasis cases and the infection rates of various animal hosts, and plotted them on a map of China using software ArcGIS10.7 (Environmental System Research Institute, Redlands, USA). Additionally, scatter plots were used to illustrate the biogeographical characteristics of regions reporting *P. westermani* and *P. skrjabini* infection. T-tests were further conducted to explore the potential differences in biogeographical characteristics between the two *Paragonimus* species.

Results

Literature selection and quality assessment

Initially, 876 publications were identified through literature search. After removing duplicates, 10,642 articles were screened based on titles and abstracts, resulting in 1,880 articles for full-text assessment. Following full-text assessments, 38 studies were ultimately included in the meta-analysis for human infections, 107 for snail infections, 172 for infections in the second intermediate host, and 22 for infections in animal reservoirs (Fig 1).

Fig 1. Flow diagram of study selection for the systematic review and meta-analysis

In the risk of bias assessment, all the studies were rated as having low to moderate bias (S1-S4 Tables). Specifically, 6 out of 38 publications for human infections, 81 out of 107 publications for snail infections, 143 out of 172 for the

second intermediate hosts, and 20 out of 22 for animal reservoirs were rated as having low bias. The most common risk identified was the lack of random selection of the sample.

Infection of *P. westermani* and *P. skrjabini* in humans

A total of 28,948 cases of human paragonimiasis have been reported in the literature, of which 2,401 cases occurred after 2010, 14,654 cases were male, and 6,089 cases were from rural areas (see Table 1). Notably, A total of 10,076 cases of infection in children or adolescents have been reported, with 8,695 cases reported before 2010 and 1,381 reported after 2010. As shown in Fig 2, human infections of *Paragonimus* have been documented in all provinces except for Tibet, Qinghai, Gansu, and Ningxia. The cases of human infection are mainly documented in provinces or municipalities in the Yangtze River Basin, including Chongqing (6,035), Zhejiang (5,324), Hubei (4,945), Sichuan (2,896), and Hunan (1,414), which together account for 71.12% of the total national cases (see Table 1). It is worth noting that after 2010, there are still a considerable number of reported cases in areas such as Chongqing (1,073) and Sichuan (595), and many other provinces and municipalities also continue to report cases.

Only a few cases differentiated whether the infection was caused by *P. westermani* and *P. skrjabini*. Cases of *P. westermani* infection are widely distributed, while *P. skrjabini* infections are primarily concentrated in more southern regions (Fig 2).

Table 1. Characteristics of human paragonimiasis cases documented in China

	Before 1990	1990-1999	2000-2009	After 2010	Total

**Province**					
Chongqing	779	984	3199	1073	6035
Zhejiang	1879	1649	1595	201	5324
Hubei	2466	989	1440	50	4945
Sichuan	640	537	1124	595	2896
Guizhou	1202	295	197	149	1843
Hunan	755	546	106	7	1414
Shaanxi	489	311	161	14	975
Liaoning	261	604	33	3	901
Fujian	490	33	341	4	868
Anhui	636	95	2	0	733
Shanghai	182	193	215	26	616
Heilongjiang	542	13	3	0	558
Jiangsu	82	369	38	40	529
Henan	176	104	44	64	388
Beijing	99	24	12	67	202
Shandong	0	176	5	0	181
Jiangxi	157	9	6	0	172
Yunnan	17	1	31	89	138
Jilin	92	3	0	2	97
Guangdong	4	23	19	15	61
Shanxi	0	14	14	0	28
Guangxi	1	23	1	0	25
Hebei	9	1	1	1	12
Hainan	0	0	3	0	3
Inner Mongoria	0	0	1	0	1
Taiwan	0	0	0	1	1
Tianjing	1	0	0	0	1
Xinjiang	0	1	0	0	1
Macao	0	0	0	0	0
Gansu	0	0	0	0	0
Ningxia	0	0	0	0	0
Qinghai	0	0	0	0	0
Tibet	0	0	0	0	0

Hong Kong	0	0	0	0	0
**Age**					
< 18	2613	2660	3422	1381	10076
≥ 18	386	454	298	229	1367
Not specified	7960	3883	4871	791	17505
**Gender**					
Male	4201	4227	4587	1639	14654
Female	1677	1932	2220	677	6506
Not specified	5081	838	1784	85	7788
**Source**					
Urban	540	263	327	52	1182
Rural	1462	1270	2992	365	6089
Not specified	8957	5464	5272	1984	21677
**Total**	10959	6997	8591	2401	28948

Fig 2. Spatial distribution of human paragonimiasis cases docummented in China.

A total of 38 studies, containing 662,003 participants, reported screening for *Paragonimus* infection in human populations (see S1 Table), with 253 confirmed cases being reported and pooled prevalence of 0.05% (95% *CI*: 0.00 - 0.12%). The heterogeneity across the studies was high (I^2 = 93.3%, Table 2; forest plot shown in S1a Fig). Subgroup analysis and the meta-regression model indicated that none of the moderators could significantly explain the heterogeneity (see S5 Table).

Table 2. Estimates of pooled prevalence and subgroup analysis of *Paragonimus* infection in humans

	**No. of data points**	**Sample size**	**No. of positive**	**Pooled prevalence, % (95% *CI*)**	**I^2, %**	**R^2, % (QM *P* value)**	**QE *P* value**
**Pathogen**	**54**	**662003**	**253**	**0.05 (0.00; 0.12)**	**93.3**	**0.00 (0.575)**	**< 0.0001**
P. westermani	30	58811	127	0.07 (0.00; 0.19)	90.1		

P. skrjabini	9	10636	22	0.04 (0.00; 0.22)	87.1		
Not specified	15	592556	104	0.03 (0.00; 0.16)	95.2		
**Year of investigation**						**4.36 (0.290)**	**< 0.0001**
Before 1990	36	57540	184	0.08 (0.01; 0.20)	88.4		
1990–1999	7	32729	35	0.08 (0.00; 0.33)	91.6		
2000–2010	5	81900	25	0.02 (0.00; 0.21)	66.7		
After 2010	6	489834	9	0.00 (0.00; 0.06)	33.0		
**Gender**						**4.17 (0.156)**	**< 0.0001**
Man	8	16340	1	0.00 (0.00; 0.11)	0		
Woman	7	5174	0	0.00 (0.00; 0.13)	0		
Not specified	39	640489	252	0.09 (0.02; 0.19)	95.2		
**Specimen**						**0.00 (0.357)**	**< 0.0001**
Sputum	38	100117	187	0.04 (0.00; 0.12)	90.3		
Stool	14	492277	22	0.03 (0.00; 0.18)	79.2		
Stool or sputum	2	69609	44	0.45 (0.04; 1.27)	98.5		

R^2 represents the proportion of true heterogeneity that can be explained by the moderator, the **QE** *P* value shows the significance of residual heterogeneity that is unaccounted for by the moderator, and the **QM** *P* value shows whether the moderator is statistically significant in explaining heterogeneity.

Infection of *P. westermani* and *P. skrjabini* in the first intermediate hosts

A total of 57 studies reported the presence of *P. westermani* infection in the first intermediate host (snails), with prevalence ranging from 0.00% to 6.72% (see S2 Table). The pooled prevalence of *P. westermani* in the first intermediate host was 0.11% (95% *CI*: 0.02－0.25%), and there was high heterogeneity across the studies (I^2= 93.6%, Table 3; forest plot is presented in S1b Fig). *Semisulcospira* spp. was identified as the most common vector of *P. westermani*, with a pooled prevalence of 0.12% (95% *CI:* 0.02－0.28%). Additionally, *Tricula* spp., *Erhaiini* spp., and *Bythinella* spp. were identified as potential vectors of *P. westermani*.

Fifty studies reported *P. skrjabini* infection in the first intermediate host, with prevalence varied from 0.00% to 14.80% (see S2 Table). The pooled prevalence of *P. skrjabini* in the first intermediate host was 0.46% (95%*CI*: 0.27—0.70%), and the heterogeneity across studies was high (I^2= 93.4%, Table 3; forest plot was shown in S1c Fig). The majority of infections in snails were reported in *Tricula* spp., with a pooled prevalence of 0.58% (95% *CI*: 0.28—0.96%). Additionally, *Pseudobythinella* spp., *Bythinella* spp., *Semisulcospira* spp., *Oncomelania* spp., *Erhaiini*spp., and *Akiyoshia* spp. were also potential vectors of *P. skrjabini*.

Spatial distribution of *P. westermani and P. skrjabini* infection in the first intermediate hosts is depicted in Fig 3. In the northeast area of China, *Semisulcospira* spp. serve as the primary transmission vectors of *Paragonimus*, and only *P. westermani* infection has been reported in this region. In more southern areas, *Semisulcospira* spp. are identified as the primary transmission vectors of *P. westermani*, while *Tricula* spp. are identified as the primary transmission vectors of *P. skrjabini* (Figs 3a and 3b).

Subgroup analysis and the meta-regression model indicated that the infection rate of *P. westermani and P. skrjabini* in the first intermediate host did not exhibit significant differences across different snail genera and time periods (see Table 3, S6 Table).

Table 3. Estimates of pooled prevalence and subgroup analysis of *Paragonimus* infection in the first intermediate hosts

	**No. of data points**	**Sample size**	**No. of positive**	**Pooled prevalence, % (95% *CI*)**	**I^2, %**	**R^2 , % (QM *P* value)**	**QE *P* value**
P. westermani	**61**	**263423**	**639**	**0.11 (0.02; 0.25)**	**93.6**		

**Year of investigation**						**3.35 (0.149)**	**< 0.0001**
Before 1990	32	120342	425	0.14 (0.01; 0.36)	91.7		
1990–1999	12	69290	52	0.04 (0.00; 0.27)	89.4		
2000–2009	11	58357	81	0.00 (0.00; 0.24)	85.6		
After 2010	6	15434	81	0.62 (0.14; 1.39)	97.3		
**Genus of snail**						**0.00 (0.637)**	**< 0.0001**
Semisulcospira	54	240158	599	0.12 (0.02; 0.28)	94.1		
Tricula	5	15918	11	0.04 (0.00; 0.41)	62.5		
Erhaiini	1	6227	26	0.42 (0.00; 2.49)	NE		
Bythinella	1	1120	3	0.27 (0.00; 2.27)	NE		
P. skrjabini	**75**	**411797**	**1343**	**0.46 (0.27; 0.70)**	**93.4**		
**Year of investigation**						**0.00 (0.678)**	**< 0.0001**
Before 1990	22	112904	428	0.52 (0.18; 1.01)	92.6		
1990–1999	14	75143	247	0.34 (0.02; 0.90)	93.9		
2000–2009	24	193038	502	0.34 (0.07; 0.75)	91.9		
After 2010	15	30712	166	0.74 (0.26; 1.43)	95.5		
**Genus of snail**						**0.00 (0.830)**	**< 0.0001**
Tricula	36	253031	643	0.58 (0.28; 0.96)	94.6		
Pseudobythinella	11	64914	340	0.57 (0.11; 1.32)	90.5		
Bythinella	10	9481	79	0.41 (0.01; 1.19)	84.7		
Semisulcospira	9	63806	178	0.06 (0.00; 0.60)	79.6		
Erhaiini	3	5789	26	0.80 (0.00; 2.66)	92.4		
Akiyoshia	3	2575	20	0.71 (0.00; 2.48)	89.5		
Oncomelania	2	10925	57	0.20 (0.00; 2.28)	0.0		
Assiminea	1	1276	0	0.00 (0.00; 1.83)	NE		

NE: not estimated; R^2 represents the proportion of true heterogeneity that can be explained by the moderator, the **QE** *P* value shows the significance of residual heterogeneity that is unaccounted for by the moderator, and the **QM** *P* value shows whether the moderator is statistically significant in explaining heterogeneity.

Fig 3. Spatial distribution of *P. westermani* and *P. skrjabini* infection in the first intermediate hosts in China. (a) *P. westermani* infection in the first intermediate hosts; (b) *P. skrjabini* infection in the first intermediate hosts

Infection of *P. westermani* and *P. skrjabini* in the second intermediate hosts

In total, 94 studies reported *P. westermani* infection in the second intermediate host (see S3 Table), with a pooled prevalence of 52.02% (95% *CI*: 44.35—59.64%) and high heterogeneity across studies (I^2 = 99.6 %, Table 4; forest plot presented in S1d Fig). Genus *Cambaroides* was identified as the primary second intermediate host for *P. westermani* in the northeastern areas of China (Fig 4a), with a pooled prevalence of 59.79% (95% *CI*: 42.65 - 75.79%; Table 4). In other areas of China, *Sinopotamon* spp. were the primary second intermediate host, with a pooled prevalence of 52.86% (95% *CI*: 43.68 — 61.94%); other freshwater crabs such as *Nanhaipotamon* spp. and *Huananpotamon* spp. could also serve as the second intermediate host (see Table 4, S3 Table).

Eighty-one studies reported *P. skrjabini* infection in the second intermediate host (see S3 Table), with a pooled prevalence of 30.37% (95% *CI*: 24.72—36.34%) and high heterogeneity across studies (I^2 = 99.8%, Table 4; forest plot presented in S1e Fig). In the northeastern region of China, only *P. westermani* has been reported in the second intermediate host, with no reports of the existence of *P. skrjabini* (see Fig 4b). The second intermediate hosts of *P. skrjabini* included crabs of the Potamidae, Lithodidae, and Parathelphusidae families. Crabs of the Potamidae family were the most common second intermediate host, with *Sinopotamon* spp. being the most significant, exhibiting a pooled prevalence of 31.53% (95% CI: 24.92% - 38.53%). Additionally, other freshwater crabs such as *Nanhaipotamon* spp., *Potamon* spp., and *Tenuilapotamon* spp. of the Potamidae family, *Somanniathelphusa* spp. of the

Parathelphusidae family, and *Malayopotamon* spp. of the Lithodidae family can also serve as the second intermediate hosts for *P. skrjabini* (see Table 4).

Subgroup analysis and the meta-regression model indicated that the infection rates of *P. westermani* and *P. skrjabini* in the second intermediate host did not exhibit significant differences among different crustacean genera, across different time periods, and with different detection methods (see Table 4, S7 Table).

Table 4. Estimates of pooled prevalence and subgroup analysis of *Paragonimus* infection in the second intermediate hosts

	**No. of data points**	**Sample size**	**No. of positive**	**Pooled prevalence, % (95% *CI*)**	***I*², %**	***R*² , % (QM *P* value)**	**QE *P* value**
P. westermani	**100**	**165276**	**40049**	**52.02 (44.35; 59.64)**	**99.6**		
**Year of investigation**						**3.27 (0.097)**	**< 0.0001**
Before 1990	44	86716	24212	62.67 (51.44; 73.25)	99.5		
1990–1999	15	66150	10488	43.24 (24.96; 62.51)	99.8		
2000–2009	22	6490	2762	41.69 (26.41; 57.82)	98.8		
After 2010	19	5920	2587	45.80 (29.06; 63.03)	99.5		
**Genus of hosts**						**0.00 (0.492)**	**< 0.0001**
Sinopotamon	70	157429	35929	52.86 (43.68; 61.94)	99.7		
Nanhaipotamon	3	175	53	26.02 (0.26; 69.67)	95.0		
Huananpotamon	2	1349	697	27.65 (0.00; 79.19)	98.8		
Malayopotamon	1	21	3	14.29 (0.00; 88.26)	NE		
Lithodes	1	72	61	84.72 (14.42; 100.00)	NE		
Eriocheir	1	85	16	18.82 (0.00; 88.53)	NE		
Cambaroides	20	5515	3110	59.79 (42.65; 75.79)	99.4		
Macrobrachium	2	630	180	28.57 (0.00; 79.70)	0.00		
**Detection method**						**0.00 (0.501)**	**< 0.0001**
Artificial digestion	55	94460	26665	50.15 (39.81; 60.49)	99.4		
Direct compression	25	8923	4135	59.75 (44.42; 74.17)	99.5		
Not specified	20	61893	9249	47.42 (30.79; 64.34)	99.7		
P. skrjabini	**109**	**198209**	**41426**	**30.37 (24.72; 36.34)**	**99.8**		

**Year of investigation**						**1.90 (0.184)**	**< 0.0001**
Before 1990	24	21578	4833	32.76 (19.69; 47.33)	99.1		
1990–1999	22	84633	6773	21.85 (11.35; 34.57)	99.8		
2000–2009	23	66211	23849	40.59 (27.31; 54.59)	99.9		
After 2010	40	25787	5971	30.13 (18.95; 42.62)	99.2		
**Genus of hosts**						**7.59 (0.065)**	**< 0.0001**
Sinopotamon	74	167883	37566	31.53 (24.92; 38.53)	99.8		
Nanhaipotamon	7	1318	480	34.30 (13.74; 58.42)	80.5		
Potamon	5	1911	458	26.03 (6.31; 53.05)	99.1		
Tenuilapotamon	3	3195	2182	27.96 (3.52; 63.48)	99.6		
Aparapotamon	3	1880	307	15.66 (0.00; 48.85)	78.0		
Bottapotamon	3	189	127	75.72 (39.60; 98.48)	96.1		
Malayopotamon	2	104	62	42.94 (5.44; 85.95)	95.8		
Huananpotamon	2	82	27	33.89 (1.83; 78.46)	0.0		
Sinolapotamon	1	3596	6	0.17 (0.00; 38.11)	NE		
Tiwaripotamon	1	3898	2	0.05 (0.00; 36.44)	NE		
Neilupotamon	1	116	6	5.17 (0.00; 58.20)	NE		
Parvuspotamon	1	223	73	32.74 (0.00; 89.38)	NE		
Potamiscus	1	24	23	95.83 (38.43; 100.00)	NE		
Tenuipotamon	1	141	38	26.95 (0.00; 85.44)	NE		
Lithodes	3	13627	69	9.41 (0.00; 39.93)	97.0		
Somanniathelphusa	1	22	0	0.00 (0.00; 47.47)	NE		
**Detection method**						**1.75 (0.135)**	**< 0.0001**
Artificial digestion	68	172632	35089	26.01 (19.38; 33.23)	99.9		
Direct compression	28	17125	4220	36.91 (25.41; 49.19)	98.3		
Not specified	13	8452	2117	40.59 (23.73; 58.66)	99.2		

NE: not estimated; R^2 represents the proportion of true heterogeneity that can be explained by the moderator, the **QE** *P* value shows the significance of residual heterogeneity that is unaccounted for by the moderator, and the **QM** *P* value shows whether the moderator is statistically significant in explaining heterogeneity.

Fig 4. Spatial distribution of *P. westermani* and *P. skrjabini* infection in the second intermediate hosts in China. (a) *P. westermani* infection in the second intermediate hosts; (b) *P. skrjabini* infection in the second intermediate hosts.

Infection of *P. westermani and P. skrjabini* in animal reservoirs

Overall, 10 studies reported *P. westermani* infection in animal reservoirs (see S4 Table), with a pooled prevalence of 21.40% (95% *CI*: 7.82—38.99%) and high heterogeneity across studies (I^2 = 94.9 %, Table 5; forest plot presented in S1g Fig). Cats (37.15% (95% *CI*: 9.61 - 69.92%)) and dogs (11.68% (95% *CI*: 0.00 - 36.56%)) were identified as the most common animal reservoirs for *P. westermani*.

Twelve studies reported *P. skrjabini* infection in animal reservoirs (see S4 Table), with a pooled prevalence of 20.31% (95% *CI*: 9.69—33.38%) and high heterogeneity across studies (I^2 = 95.2 %, Table 5; forest plot presented in S1f Fig). Similar to *P. westermani*, cats (36.35% (95% *CI*: 20.74–53.51 %)) and dogs (5.79% (95% *CI*: 0.00–23.03 %)) were identified the most common animal reservoirs for *P. skrjabini*.

Subgroup analysis and meta-regression models indicated that animal categories, lifestyle (wild or domestic), or detection methods could significantly explain the observed heterogeneity (see Table 5, S8 Table).

Table 5. Estimates of pooled prevalence and subgroup analysis of *Paragonimus* infection in animal reservoirs

	**No. of data points**	**Sample size**	**No. of positive**	**Pooled prevalence, % (95% *CI*)**	**I^2, %**	**R^2, % (QM *P* value)**	**QE *P* value**
P. westermani	**13**	**1353**	**307**	**21.40 (7.82; 38.99)**	**94.9**		
**Year of investigation**						**5.40 (0.266)**	**< 0.0001**
Before 1990	7	999	275	34.00 (12.82; 59.07)	94.0		
1990–1999	3	269	25	12.25 (0.00; 46.27)	95.2		
After 2010	3	85	7	6.37 (0.00; 37.97)	75.4		

**Family of hosts**						**0.00 (0.609)**	**< 0.0001**
Canidae	6	936	210	11.68 (0.00; 36.56)	96.2		
Felidae	5	299	74	37.15 (9.61; 69.92)	96.1		
Viverridae	1	66	13	19.70 (0.00; 85.85)	NE		
Mustelidae	1	52	10	19.23 (0.00; 85.87)	NE		
**Life style**						**0.00 (0.702)**	**< 0.0001**
Domestic	10	1214	274	24.70 (4.93; 40.41)	96.1		
Wild	3	139	33	22.57 (1.18; 68.23)	69.3		
**Detection method**						**0.00 (0.545)**	**< 0.0001**
Sedimentation	2	55	7	12.44 (0.00; 60.11)	42.5		
Direct compression	2	54	22	41.81 (2.41; 89.04)	0.00		
Kato-Katz	1	30	0	0.00 (0.00; 51.65)	NE		
Not specified	8	1214	278	23.59 (6.13; 47.41)	96.6		
P. skrjabini	**20**	**1067**	**180**	**20.31 (9.69; 33.38)**	**95.2**		
**Year of investigation**						**10.34 (0.168)**	**<0.0001**
Before 1990	5	199	53	30.53 (8.60; 58.19)	94.5		
1990–1999	5	408	17	3.52 (0.00; 21.03)	91.3		
2000–2009	5	167	56	30.31 (8.36; 58.09)	83.4		
After 2010	5	293	54	23.88 (4.94; 50.39)	96.3		
**Family of hosts**						**26.53 (0.046)**	**< 0.0001**
Felidae	11	433	146	36.35 (20.74; 53.51)	93.7		
Canidae	5	319	20	5.79 (0.00; 23.03)	79.5		
Muridae	1	223	0	0.00 (0.00; 29.15)	NE		
Viverridae	1	43	8	18.60 (0.00; 72.12)	NE		
Suidae	1	21	0	0.00 (0.00; 39.21)	NE		
Mustelidae	1	28	6	21.43 (0.00; 76.78)	NE		
**Life style**						**20.34 (0.018)**	**< 0.0001**
Domestic	11	480	130	33.12 (17.50; 50.78)	95.3		
Wild	9	587	50	8.09 (0.40; 22.06)	92.3		
**Detection method**						**25.37 (0.029)**	**< 0.0001**
Direct compression	6	231	90	45.69 (23.38; 68.90)	92.7		
Sedimentation	9	542	65	13.88 (3.16; 29.65)	94.9		
Kato-Katz	2	194	5	1.28 (0.00; 24.62)	70.6		
Not specified	3	100	20	15.93 (0.05; 46.32)	89.7		

NE: not estimated; $\boldsymbol{R^2}$ represents the proportion of true heterogeneity that can be explained by the moderator, the **QE** *P* value shows the significance of residual heterogeneity that is unaccounted

for by the moderator, and the **QM** *P* value shows whether the moderator is statistically significant in explaining heterogeneity.

Publish bias and sensitivity analysis

Asymmetry in the funnel plots and the results of Egger's test indicated the presence of publication bias (see S2 Fig). The sensitivity analysis results demonstrated that the pooled prevalence estimate did not change significantly after the removal of outlier data points or data points with small sample sizes (95% *CI* overlapped; see S9 Table).

Biogeographical characteristics of *P. westermani* and *P. skrjabini* infections

To investigate the biogeographical characteristics of *Paragonimus* occurrences, we created scatter plots using the climate features of *P. westermani* and *P. skrjabini* endemic sites and 1000 random points. The results indicate that, compared to random points, endemic sites of *P. westermani* and *P. skrjabini* are mainly distributed in regions with lower altitude and higher temperature and precipitation (see S10 Table and Fig 5). Specifically, endemic sites of *P. westermani* are predominantly distributed in areas with an altitude below 1166.0m, annual temperature above 1.0°C, annual precipitation above 541.0mm, mean temperature of the warmest quarter above 18.3°C, and precipitation of the warmest quarter above 304.0mm. On the other hand, endemic sites of *P. skrjabini* are distributed in areas with altitude below 2188.0m, annual temperature above 10.9°C, annual precipitation above 578.0mm, mean temperature of the warmest quarter above 19.5°C, and precipitation of the warmest quarter above 257.0mm. When comparing the two *Paragonimus* species, the endemic points of *P.*

westermani have lower altitudes (below 1166.0m for *P. westermani*; 2188.0m for *P. skrjabini*) and lower mean temperature of the coldest quarter (above -20.1°C for *P. westermani*; -0.8°C for *P. skrjabini*).

Fig 5. Environmental characteristics of areas with reported *Paragonimus* infections in China.

Discussion

In this study, we summarized the infection status and geographical distribution of *P. westermani* and *P. skrjabini* in humans and animal hosts in China. Our findings indicate that *Paragonimus* infection is widely distributed and remains prevalent in China, with variations in the transmission vectors, second intermediate hosts, and geographical distribution between *P. westermani* and *P. skrjabini*. Furthermore, environmental factors such as temperature and precipitation may influence the distribution of *Paragonimus*.

After years of educational efforts, the reported number of human paragonimiasis cases has significantly decreased in most areas of China (see Table 1). However, it is noteworthy that after 2010, a considerable number of reported cases persist in areas such as Chongqing (1073) and Sichuan (595), with many other provinces and municipalities also continuing to report cases, highlighting the need for ongoing control efforts against paragonimiasis. Another notable issue is the significant involvement of children and adolescents in paragonimiasis cases, both before and after 2010 [22-24]. In certain endemic areas, particularly in rural or mountainous regions, practices such as local children drinking untreated water and consuming

undercooked shrimp and crab are more common among children compared to adults [25, 26], underscoring the necessity for enhanced health education on paragonimiasis in schools in key areas. Additionally, human infection may be more widespread and underestimated due to a lack of training of health workers to identify paragonimiasis and a deficient case-reporting system [27].

The distribution regions of *P. westermani* and *P. skrjabini* in China exhibit both differences and overlaps. In the northeastern areas of China, only *P. westermani* has been documented, while in the southern part of China, both species coexist. The difference in the distribution of the two *Paragonimus* species is likely due to variations in their second intermediate hosts. Specifically, *Sinopotamon*, primarily distributed in the southern part of China, serves as the main second intermediate host for both *P. westermani* and *P. skrjabini* [28, 29]. On the other hand, *Cambaroides*, which inhabits the northeastern region of China, can only serve as the second intermediate host for *P. westermani* [30]. On the other hand, *Cambaroides,* which inhabits the northeastern region of China, can only serve as the second intermediate host for P. westermani [30]. It has been reported that *P. westermani* and *P. skrjabini* share some common intermediate hosts, such as *Semisulcospira*, *Tricula*, *Erhaiini*, and *Bythinella* in the first intermediate host, and *Huananpotamon* in the second intermediate host [31, 32]. Additionally, the cercariae and metacercariae of *P. westermani* and *P. skrjabini* are morphologically similar[33]. Therefore, in areas where the two *Paragonimus* species overlap, there may be misclassification when detecting the infection in intermediate hosts. To accurately differentiate between the different *Paragonimus* species, nucleic acid detection is recommended to be conducted simultaneously in epidemiological surveys.

The infection rates of *Paragonimus* in intermediate hosts exhibit significant

variation. In the first intermediate host, the infection rate of *P. westermani* ranged from 0.00% to 6.72%, while the infection rate of *P. skrjabini* ranged from 0.00% to 14.80% (see S2 Table). In the second intermediate host, the infection rate ranged from 0% to 100% (see S3 Table). None of the known moderators, including the taxonomic category of the intermediate host, year of survey, and detection methods, can significantly explain the heterogeneity across studies (see S6 and S7 Tables). Therefore, it is necessary to conduct random sampling surveys in different regions to further understand the factors that influence the infection rates of *Paragonimus* in intermediate hosts.

In many regions of China, it is common for residents to consume marinated or drunken crabs in their raw state [4, 34]. However, the methods of salting and soaking in alcohol are not completely effective in killing the metacercariae [35, 36]. Another prevalent practice is the consumption of freshwater crabs and crayfish through stir-frying, but inadequate heating may not fully eliminate the parasites [37, 38]. Human infection occurs through the consumption of inadequately cooked freshwater crustaceans containing the infective metacercariae. Given the persistently high infection rates of *Paragonimus* in the second intermediate host (with a pooled prevalence of 52.02% (95% CI: 42.65 - 75.79%) for *P. westermani* and 30.37% (95% CI: 24.72 - 36.34%) for *P. skrjabini*; see Table 4), and the continued popularity of consuming raw or undercooked freshwater crustaceans in many areas of China, paragonimiasis remains a significant public health threat to the Chinese population.

The analysis of biogeographical characteristics revealed that temperature and precipitation might influence the distribution of *Paragonimus* (see Fig. 4). Temperature may affect the distribution of *Paragonimus* by influencing the survival of the intermediate host (snails and crustaceans) or by affecting the development of

Paragonimus. For example, research by Hu et al. indicates that the development of the eggs of *P. heterotremus* is closely related to the external temperature [39]. Development is slow or even halted at temperatures below 12°C, and does not occur at temperatures above 37°C. Chiu has found that the optimum temperature for the development of *P. iloktsuensis* in *T. chiui* is 22 to 30°C [40]. Our study results indicate that, compared to *P. skrjabini*, *P. westermani* can survive in regions with lower temperatures, such as northeastern China (see Fig. 5), suggesting that *P. westermani* exhibits great tolerance to low temperatures. Similarly, Fan and colleagues have found that metacercariae of *P. westermani* can still develop into mature worms in rats after storage at 4°C for up to 234 days [41].

Paragonimus infections have been predominantly documented in eastern China. This geographical distribution is closely associated with water supply, with precipitation playing a crucial role in the distribution of aquatic snails and crustaceans [42], both of which are integral to the *Paragonimus* life cycle. The higher levels of precipitation in eastern China create environments that are more conducive to the survival and proliferation of intermediate hosts, thereby increasing the risk of *Paragonimus* infections in these areas [43].

In this study, we pooled studies from numerous sites to achieve a relatively large sample size to summarize the infection rate of *P. westermani* and *P. skrjabini* in humans, intermediate hosts, and animal reservoirs. However, several limitations of our study should be considered. Firstly, the absence of literature reporting *Paragonimus* infections in certain areas does not necessarily indicate that *Paragonimus* infections do not exist there; it may be due to a lack of research in those areas or unpublished research findings. Secondly, significant heterogeneity was detected across studies, and most of the heterogeneity could not be explained by

known moderators. Lastly, publication bias exists in this study, which may cause bias in the estimates of pooled prevalence. Therefore, the results of our study should be interpreted with caution. Despite these limitations, our study systematically summarizes the infection status of *P. westermani* and *P. skrjabini* in humans, intermediate hosts, and animal reservoirs in China, and elucidates their spatial distribution. The findings may provide valuable insights for the control of paragonimiasis in China.

Conclusions

Paragonimus infection remains widely distributed and prevalent in China, with children and adolescents at high risk in endemic areas. Variations exist in the intermediate hosts and geographical distribution of *P. westermani* and *P. skrjabini* infections in China. *P. skrjabini* infections are predominantly concentrated in more southern regions compared to *P. westermani*. Additionally, temperature and precipitation may influence the distribution of *P. westermani* and *P. skrjabini*.

Financial Disclosure Statement

This research was funded by the Shandong Provincial Natural Science Foundation (https://cloud.kjt.shandong.gov.cn/) (grant no. ZR2019MH093, ZR2023MH313), the Shandong Provincial Youth Innovation Team Development Plan of Colleges and Universities (http://edu.shandong.gov.cn/) (grant no. 2019-6-156, Lu-Jiao), the National Parasite Resource Bank (https://www.most.gov.cn/index.html) (grant no. NPRC-2019-194-30), the Quality Education Teaching Resources Project of Shandong

Province and Weifang Medical University (http://edu.shandong.gov.cn/) (grant no. SDYAL2022152, 22YZSALK01, 23YJSALK01), Joint Research Program of China National Center for Food Safety Risk Assessment (https://cfsa.net.cn/) (grant no. LH2022GG08, LH2022GG02), and the National Natural Science Foundation of China (https://www.nsfc.gov.cn/) (grant no. 81902905).

The funders had no role in study design, data collection and analysis, decision to publish, or preparation of the manuscript.

Author Contributions

Conceptualization: Lan-Hua Li, Yan Lu, Fu-Yan Shi.

Data curation: Kai Liu, Yuan-Chao Sun, Rui-Tai Pan, Ao-Long Xu, Han Xue.

Formal analysis: Kai Liu, Na Tian, Jin-Xin Zheng.

Supervision: Lan-Hua Li, Yan Lu, Fu-Yan Shi.

Visualization: Rui-Tai Pan, Ao-Long Xu, Han Xue, Jin-Xin Zheng.

Writing – original draft: Kai Liu, Yuan-Chao Sun.

Writing – review & editing: Jin-Xin Zheng, Fu-Yan Shi, Yan Lu, Lan-Hua Li.

References

1. Singh TS, Sugiyama H, Rangsiruji A. *Paragonimus* & paragonimiasis in India. Indian J Med Res. 2012;136(2):192-204.

2. Yoshida A, Doanh PN, Maruyama H. *Paragonimus* and paragonimiasis in Asia: An update. Acta Trop. 2019;199:105074, doi:10.1016/j.actatropica.2019.105074.

3. Keiser J, Utzinger J. Emerging foodborne trematodiasis. Emerg Infect Dis. 2005;11(10):1507-14, doi:10.3201/eid1110.050614.

4. Liu Q, Wei F, Liu W, Yang S, Zhang X. Paragonimiasis: an important food-borne zoonosis in China. Trends Parasitol. 2008;24(7):318-23, doi:10.1016/j.pt.2008.03.014.

5. Blair D, Xu ZB, Agatsuma T. Paragonimiasis and the genus *Paragonimus*. Adv Parasitol. 1999;42:113-222, doi:10.1016/s0065-308x(08)60149-9.

6. Feng Y, Fürst T, Liu L, Yang GJ. Estimation of disability weight for paragonimiasis: a systematic analysis. Infect Dis Poverty. 2018;7(1):110, doi:10.1186/s40249-018-0485-5.

7. Lu XT, Gu QY, Limpanont Y, Song LG, Wu ZD, Okanurak K, et al. Snail-borne parasitic diseases: an update on global epidemiological distribution, transmission interruption and control methods. Infect Dis Poverty. 2018;7(1):28, doi:10.1186/s40249-018-0414-7.

8. Huang YX, Zhang S, Sheng Y, Liang J, Wang J, Liu XW, et al. Investigation of *Paragonimus westermani* host in Kuandian County of Liaoning province. J Med Pest Control. 2016;32(04):420-1 (in Chinese).

9. Vélez I, Velásquez LE, Vélez ID. Morphological description and life cycle of *Paragonimus* sp. (Trematoda: Troglotrematidae): causal agent of human paragonimiasis in Colombia. J Parasitol. 2003;89(4):749-55, doi:10.1645/ge-2858.

10. Wilairatana P, Kotepui KU, Mala W, Wangdi K, Kotepui M. Prevalence, probability, and characteristics of malaria and filariasis co-infections: A systematic review and meta-analysis. PLoS Negl Trop Dis. 2022;16(10):e0010857, doi:10.1371/journal.pntd.0010857.

11. Lai MC, Kassee C, Besney R, Bonato S, Hull L, Mandy W, et al. Prevalence of co-occurring mental health diagnoses in the autism population: a systematic review and meta-analysis. Lancet Psychiatry. 2019;6(10):819-29, doi:10.1016/s2215-0366(19)30289-5.

12. Hoy D, Brooks P, Woolf A, Blyth F, March L, Bain C, et al. Assessing risk of bias in prevalence studies: modification of an existing tool and evidence of interrater agreement. J Clin Epidemiol. 2012;65(9):934-9, doi:10.1016/j.jclinepi.2011.11.014.

13. Gong QL, Chen Y, Tian T, Wen X, Li D, Song YH, et al. Prevalence of bovine tuberculosis in dairy cattle in China during 2010-2019: A systematic review and meta-analysis. PLoS Negl Trop Dis. 2021;15(6):e0009502, doi:10.1371/journal.pntd.0009502.

14. Li R, Li W, Lun Z, Zhang H, Sun Z, Kanu JS, et al. Prevalence of metabolic syndrome in Mainland China: a meta-analysis of published studies. BMC Public Health. 2016;16:296, doi:10.1186/s12889-016-2870-y.

15. Faustino R, Faria M, Teixeira M, Palavra F, Sargento P, do Céu Costa M. Systematic review and meta-analysis of the prevalence of coronavirus: One health approach for a global strategy. One Health. 2022;14:100383, doi:10.1016/j.onehlt.2022.100383.

16. Velayudhan L, McGoohan K, Bhattacharyya S. Safety and tolerability of natural and synthetic cannabinoids in adults aged over 50 years: A systematic review and meta-analysis. PLoS Med. 2021;18(3):e1003524, doi:10.1371/journal.pmed.1003524.

17. Zhu JJ, Lu DL, Zhang WD. Effects of gaps on regeneration of woody plants: a meta-analysis. J Forestry Res. 2014;25(3):501-10, doi:10.1007/s11676-014-0489-3.

18. Al Maqbali M, Al Sinani M, Al-Lenjawi B. Prevalence of stress, depression, anxiety and sleep disturbance among nurses during the COVID-19 pandemic: A systematic review and meta-analysis. J Psychosom Res. 2021;141:110343, doi:10.1016/j.jpsychores.2020.110343.

19. Irwig L, Macaskill P, Berry G, Glasziou P. Bias in meta-analysis detected by a simple, graphical test. Graphical test is itself biased. Bmj. 1998;316(7129):470; author reply -1.

20. Riquetti NB, Mello CR, Beskow S, Viola MR. Rainfall erosivity in South America: Current patterns and future perspectives. Sci Total Environ. 2020;724:138315, doi:10.1016/j.scitotenv.2020.138315.

21. Amoakoh AO, Aplin P, Awuah KT, Delgado-Fernandez I, Moses C, Alonso CP, et al. Testing the Contribution of Multi-Source Remote Sensing Features for Random Forest Classification of the Greater Amanzule Tropical Peatland. Sensors (Basel). 2021;21(10), doi:10.3390/s21103399.

22. Li L, Zhang Y, Zhu J, Zhai X, Cai J, He L, et al. Intracranial Pseudoaneurysm Caused by Cerebral Paragonimiasis in Pediatric Patients. Pediatr Neurol. 2020;109:47-51, doi:10.1016/j.pediatrneurol.2020.03.018.

23. Xu HZ, Tang LF, Zheng XP, Chen ZM. Paragonimiasis in chinese children: 58 cases analysis. Iran J Pediatr. 2012;22(4):505-11.

24. Qian M, Li F, Zhang Y, Qiao Z, Shi Y, Shen J. A retrospective clinical analysis of pediatric paragonimiasis in a Chinese children's hospital from 2011 to 2019. Scientific Reports. 2021;11(1):2005, doi:10.1038/s41598-021-81694-7.

25. Gong Z, Miao R, Shu M, Zhu Y, Wen Y, Guo Q, et al. Paragonimiasis in Children in Southwest China: A retrospective case reports review from 2005 to 2016. Medicine. 2017;96(25):e7265, doi:10.1097/md.0000000000007265.

26. Devi KR, Narain K, Bhattacharya S, Negmu K, Agatsuma T, Blair D, et al. Pleuropulmonary paragonimiasis due to *Paragonimus* heterotremus: molecular diagnosis, prevalence of infection and clinicoradiological features in an endemic area of northeastern India. Trans R Soc Trop Med Hyg. 2007;101(8):786-92, doi:10.1016/j.trstmh.2007.02.028.

27. Rabone M, Wiethase J, Clark PF, Rollinson D, Cumberlidge N, Emery AM. Endemicity of *Paragonimus* and paragonimiasis in Sub-Saharan Africa: A systematic review and mapping reveals stability of transmission in endemic foci for a multi-host parasite system. PLoS Negl Trop Dis. 2021;15(2):e0009120, doi:10.1371/journal.pntd.0009120.

28. Shih HT, Huang C, Ng PK. A re-appraisal of the widely-distributed freshwater crab genus *Sinopotamon* Bott, 1967, from China, with establishment of a new genus (Crustacea: Decapoda: Potamidae). Zootaxa. 2016;4138(2):309-31, doi:10.11646/zootaxa.4138.2.5.

29. Huang C, Shih HT, Mao SY. *Yuebeipotamon calciatile*, a new genus and new species of freshwater crab from southern China (Crustacea, Decapoda, Brachyura, Potamidae). Zookeys. 2016(615):61-72, doi:10.3897/zookeys.615.9964.

30. Bao J, Xing Y, Feng C, Kou S, Jiang H, Li X. Acute and sub-chronic effects of copper on survival, respiratory metabolism, and metal accumulation in *Cambaroides dauricus*. Scientific Reports. 2020;10(1):16700, doi:10.1038/s41598-020-73940-1.

31. Chen JQ, Liu SH, Luo J, Cai MR, Cheng YZ. Investigation on freshwater crab populations and Paragonimus infections in the Minjiang River basin along the middle section

of Wuyi Mountain. Chin J Schisto Control. 2021;33(6):590-9, doi:10.16250/j.32.1374.2021154 (in Chinese).

32. Cheng YZ, Li LS, Lin GH, Zhou PC, Jiang DW, Fang YY, et al. Survey on the foci of *Paragonimus* in Youxi, Yongtai and Pinghe Counties of Fujian Province. Chin J Parasitol Parasit Dis. 2010;28(6):406-10 (in Chinese).

33. Li BW, McNulty SN, Rosa BA, Tyagi R, Zeng QR, Gu KZ, et al. Conservation and diversification of the transcriptomes of adult *Paragonimus westermani* and *P. skrjabini*. Parasit Vectors. 2016;9(1):497, doi:10.1186/s13071-016-1785-x.

34. Tidman R, Kanankege KST, Bangert M, Abela-Ridder B. Global prevalence of 4 neglected foodborne trematodes targeted for control by WHO: A scoping review to highlight the gaps. PLoS Negl Trop Dis. 2023;17(3):e0011073, doi:10.1371/journal.pntd.0011073.

35. Sasaki J, Matsuoka M, Kinoshita T, Horii T, Tsuneyoshi S, Murata D, et al. A Cluster of Paragonimiasis with Delayed Diagnosis Due to Difficulty Distinguishing Symptoms from Post-COVID-19 Respiratory Symptoms: A Report of Five Cases. Medicina (Kaunas). 2023;59(1), doi:10.3390/medicina59010137.

36. Sharma OP. The man who loved drunken crabs. A case of pulmonary paragonimiasis. Chest. 1989;95(3):670-2, doi:10.1378/chest.95.3.670.

37. Chen WQ, Deng Y, Zhang YL, Ai L, Chen JX, Lin XM, et al. A case of group infections with *Paraginimus* species in Henan, Central China. Acta Trop. 2020;202:105111, doi:10.1016/j.actatropica.2019.105111.

38. Peng X, Zhang J, Zhang J, Wang Y, Zhang X. Incidence of paragonimiasis in Chongqing China: a 6-year retrospective case review. Parasitology. 2018;145(6):792-6, doi:10.1017/s003118201700172x.

39. Hu W. Studies on the life cycle of *Paragonimus* heterotremus. Chin J Parasitol Parasit Dis. 1998;16(5):347-52 (in Chinese).

40. Chiu JK. Tríenla chiui Habe et Miyazaki, 1962: a snail host for *Paragonimus* iloktsuenensis Chen, 1940 in Taiwan. Jap J Para-sitol. 1965;14(3):269-80.

41. Fan PC, Lu H, Lin LH. Experimental infection of *Paragonimus westermani* in mice and rats. Korean J Parasitol. 1993;31(2):91-7, doi:10.3347/kjp.1993.31.2.91.

42. Yin Y, He Q, Pan X, Liu Q, Wu Y, Li X. Predicting Current Potential Distribution and the Range Dynamics of *Pomacea canaliculata* in China under Global Climate Change. Biology (Basel). 2022;11(1), doi:10.3390/biology11010110.

43. Chen Y, Zhu M. Spatiotemporal Evolution and Driving Mechanism of "Production-Living-Ecology" Functions in China: A Case of Both Sides of Hu Line. Int J Environ Res Public Health. 2022;19(6), doi:10.3390/ijerph19063488.

Supporting information

S1Table. Publications reporting *Paragonimus* infection in humans.

S2Table. Publications reporting *Paragonimus* infection in the first intermediate hosts.

S3Table. Publications reporting *Paragonimus* infection in the second intermediate hosts.

S4Table. Publications reporting *Paragonimus* infection in animal reservoirs.

S5Table. Multivariable meta-regression analyses for *Paragonimus* infection in humans.

S6Table. Multivariable meta-regression analyses for *Paragonimus* infection in the first intermediate hosts.

S7Table. Multivariable meta-regression analyses for *Paragonimus* infection in the second intermediate hosts.

S8Table. Multivariable meta-regression analyses for *Paragonimus* infection in animal reservoirs.

S9Table. Sensitivity analysis of the pooled prevalence of *Paragonimus* in humans, the first intermediate hosts, the second intermediate hosts, and animal reservoirs.

S10Table. Environmental characteristics of areas with reported *P. westermani* and *P. skrjabini* infections in China.

S1Fig. Forest plots of prevalence of *Paragonimus* species in humans, the first intermediate host, the second intermediate host, and animal reservoirs. (a) *Paragonimus* in humans; (b) *P. westermani* in the first intermediate host; (c) *P. skrjabini* in the first intermediate host; (d) *P. westermani* in the second intermediate host; (e) *P. skrjabini* in the second intermediate host; (f) *P. skrjabini* in animal reservoir; (g) *P. westermani* in animal reservoir.

S2Fig. Funnel plot for assessing publication bias in studies reporting prevalence of *Paragonimus* species in humans, the first intermediate host, the second intermediate host, and animal reservoirs (a) *Paragonimus* in humans; (b) *P. skrjabini* in the first intermediate host; (c) *P. westermani* in the first intermediate host; (d) *P. skrjabini*

in the second intermediate host; (e) *P. westermani* in the second intermediate host; (f) *P. skrjabini* in animal reservoir; (g) *P. westermani* in animal reservoir.

Figures and Tables

Fig 1. Flow diagram of study selection for the systematic review and meta analysis.

Fig 2. Spatial distribution of human paragonimiasis cases docummented in China.

Fig 3. Spatial distribution of *P. westermani* and *P. skrjabini* infection in the first intermediate hosts in China. (a) *P. westermani* infection in the first intermediate hosts; (b) *P. skrjabini* infection in the first intermediate hosts;

Fig 4. Spatial distribution of *P. westermani* and *P. skrjabini* infection in the second intermediate hosts in China. (a) *P. westermani* infection in the second intermediate hosts; (b) *P. skrjabini* infection in the second intermediate hosts.

Fig 5. Environmental characteristics of areas with reported *Paragonimus* infections in China.

Table 1. Characteristics of human paragonimiasis cases documented in China.

Table 2. Estimates of pooled prevalence and subgroup analysis of *Paragonimus* in humans.

Table 3. Estimates of pooled prevalence and subgroup analysis of *Paragonimus* in the first intermediate host.

Table 4. Estimates of pooled prevalence and subgroup analysis of *Paragonimus* in the second intermediate host.

Table 5. Estimates of pooled prevalence and subgroup analysis of *Paragonimus* in animal reservoirs.

Data reporting

The data that supports the findings of this study are available in the supplementary material of this article.

Fig 1. Flow diagram of study selection for the systematic review and meta analysis.

Click here to access/download;Figure;Fig 1.tif

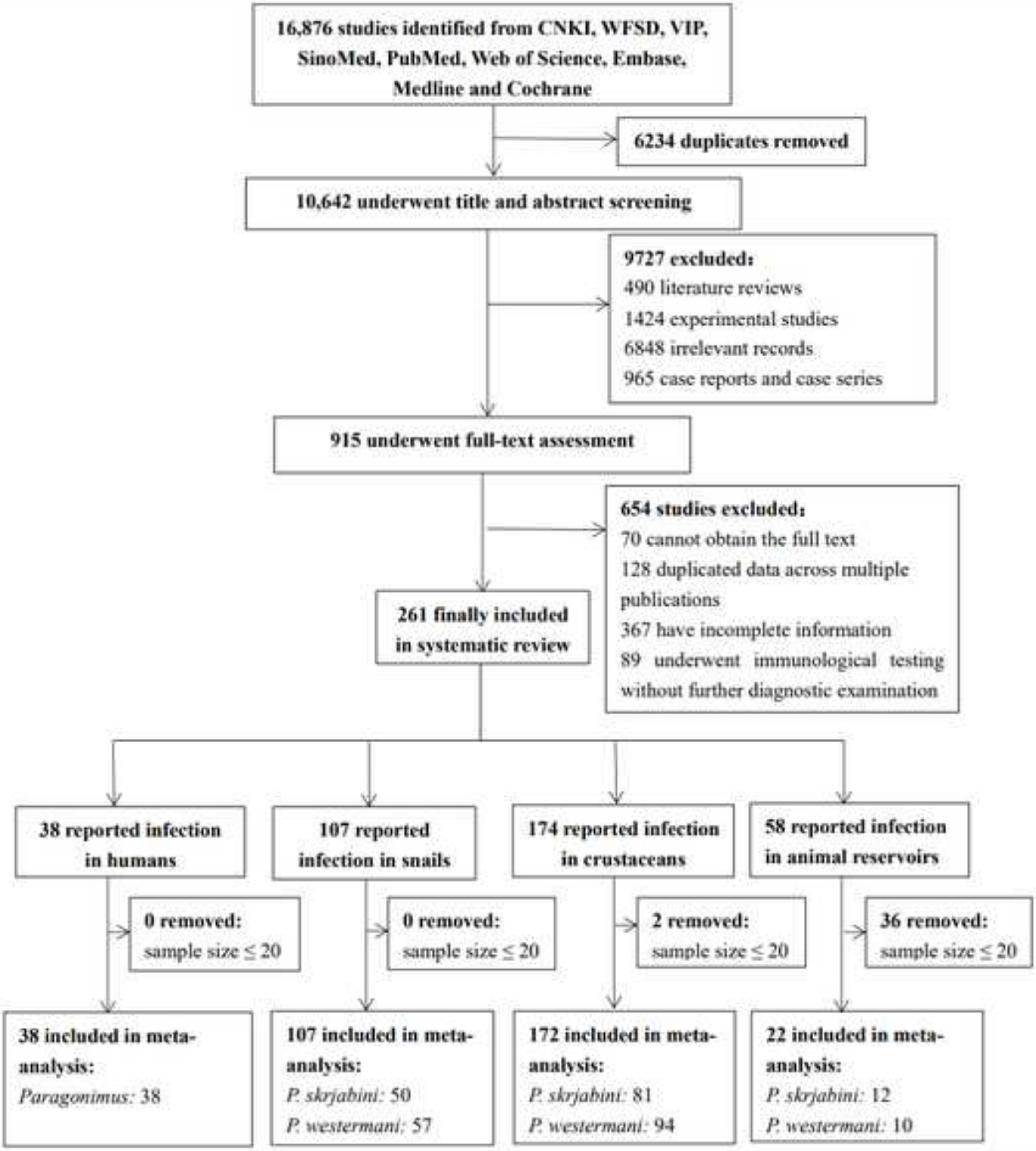

Fig 2. Spatial distribution of human paragonimiasis cases documented in China.

Click here to access/download;Figure;Fig 2.eps

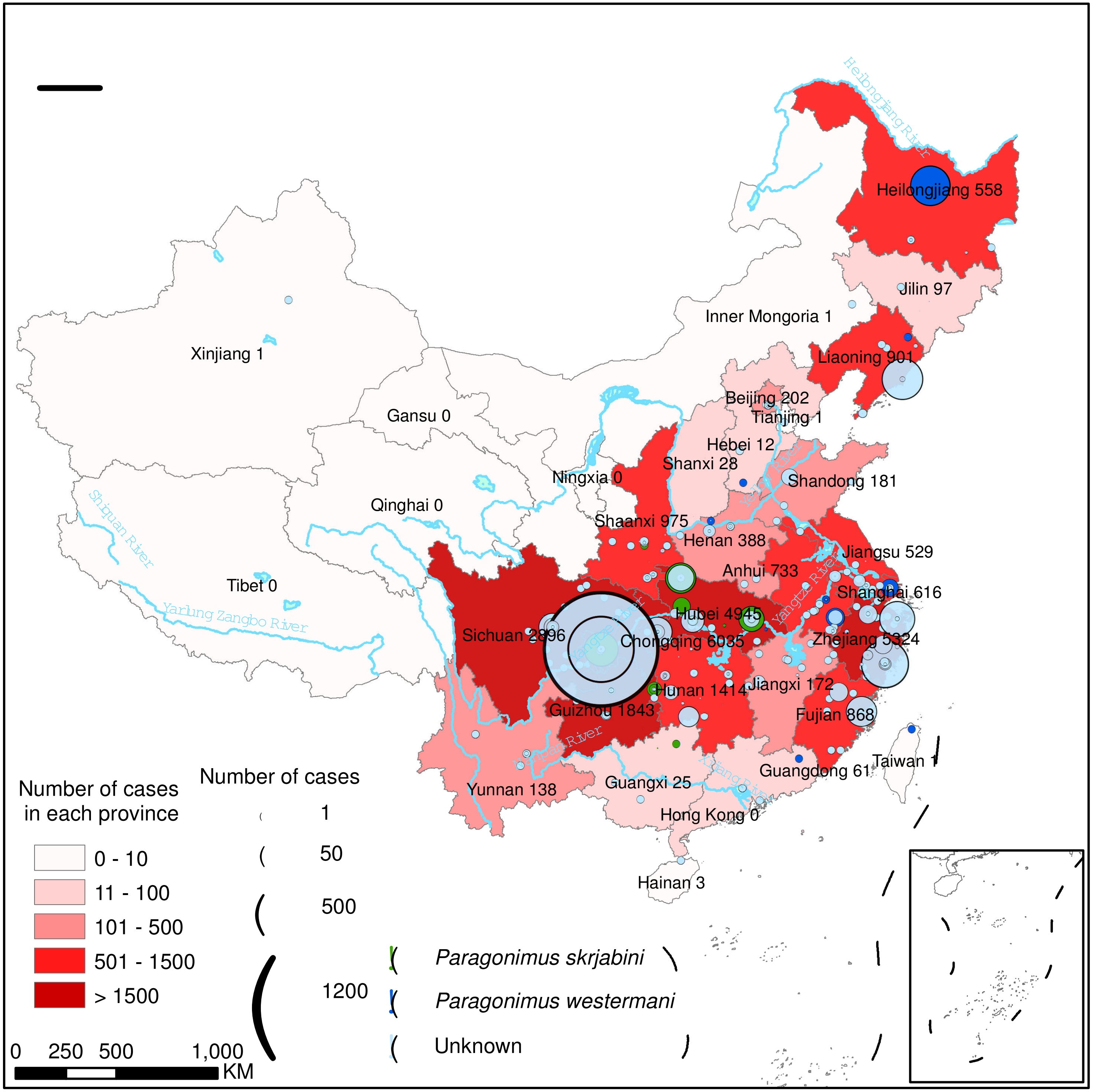

Fig 3a. Spatial distribution of P. westermani infection in the first intermediate hosts.

Click here to access/download;Figure;Fig 3a.eps

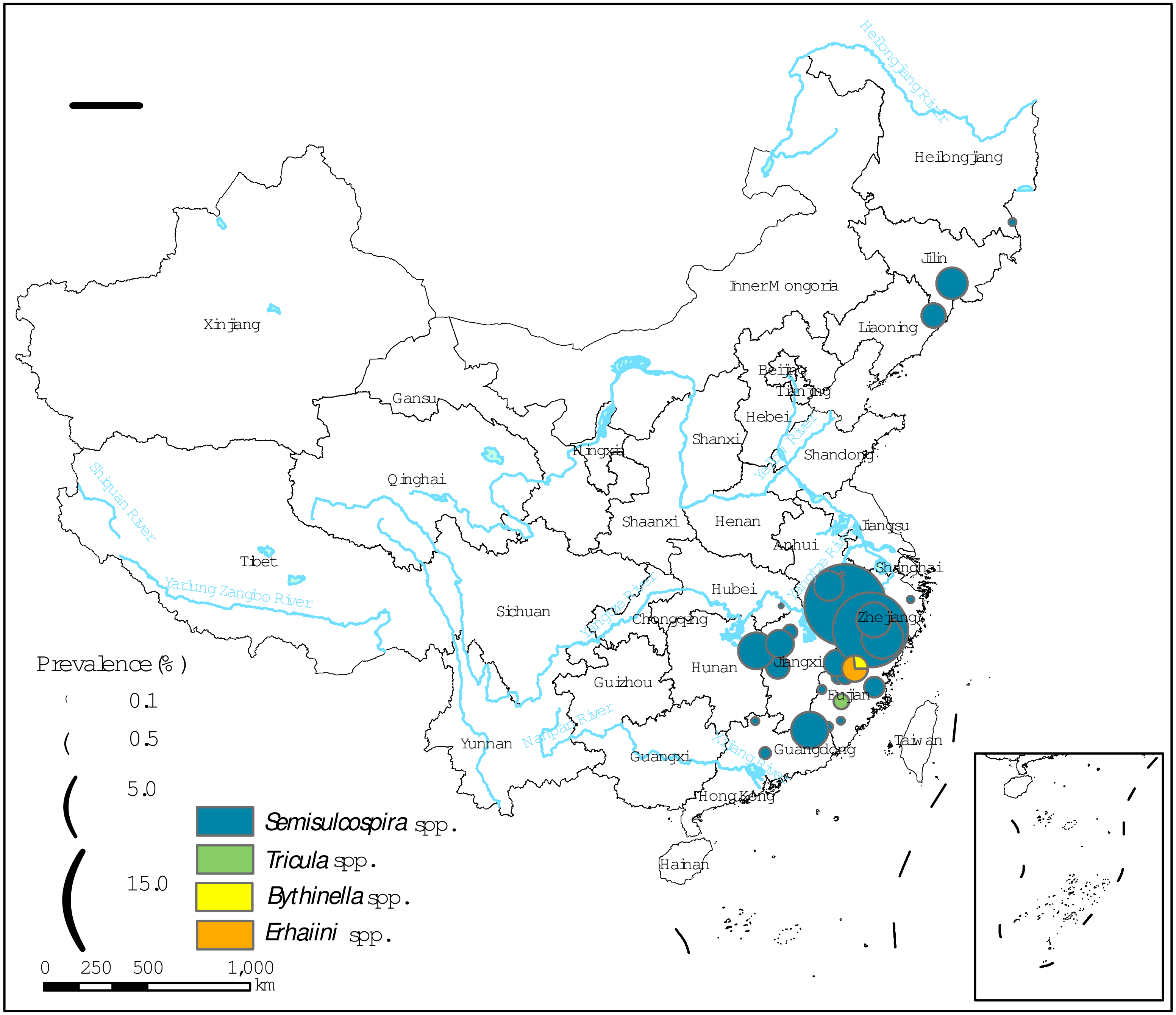

Fig 3b. Spatial distribution of P. skrjabini infection in the first intermediate hosts.

Click here to access/download;Figure;Fig 3b.eps

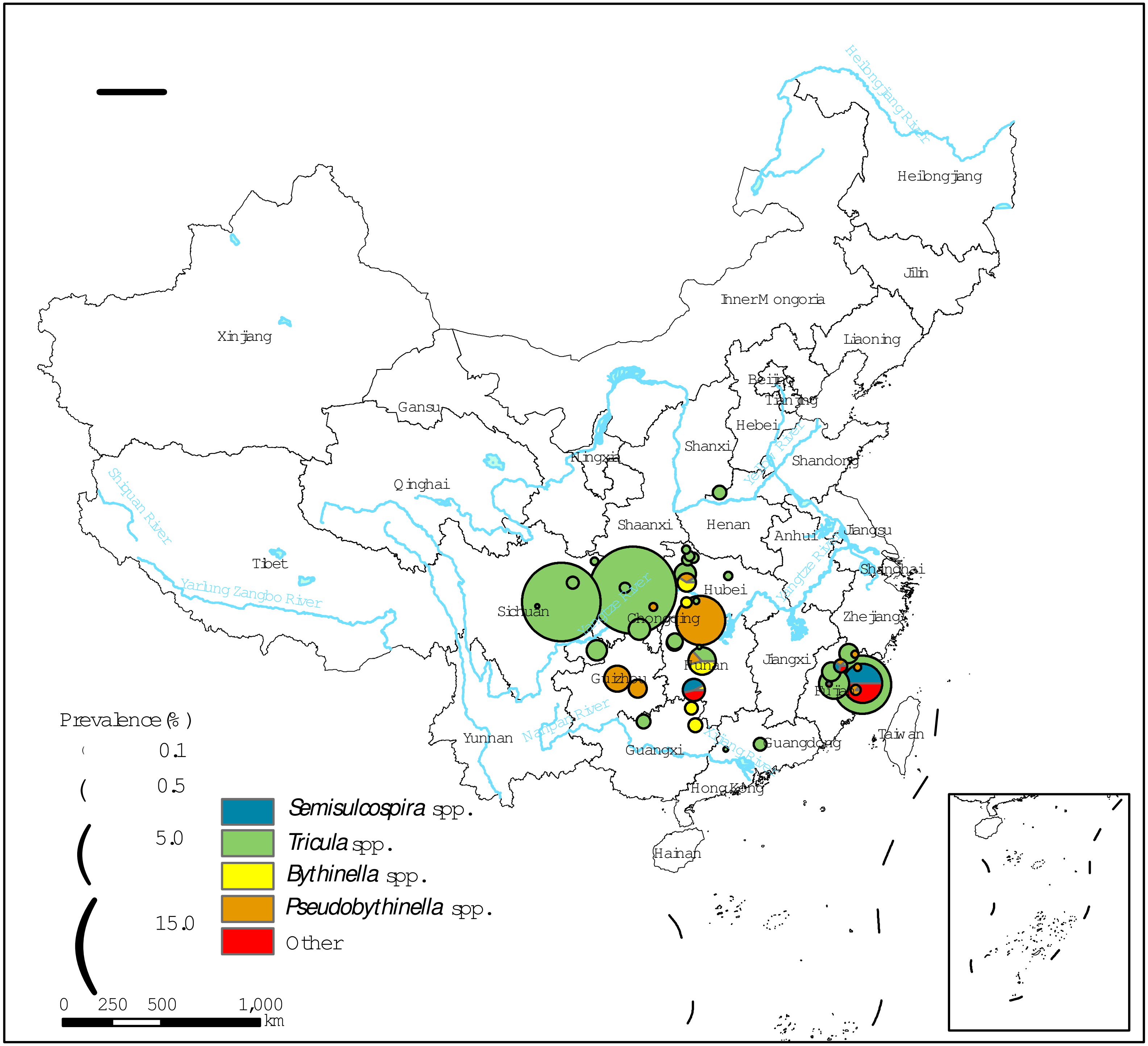

Fig 4a. Spatial distribution of P. westermani infection in the second intermediate hosts.

Click here to access/download;Figure;Fig 4a.eps

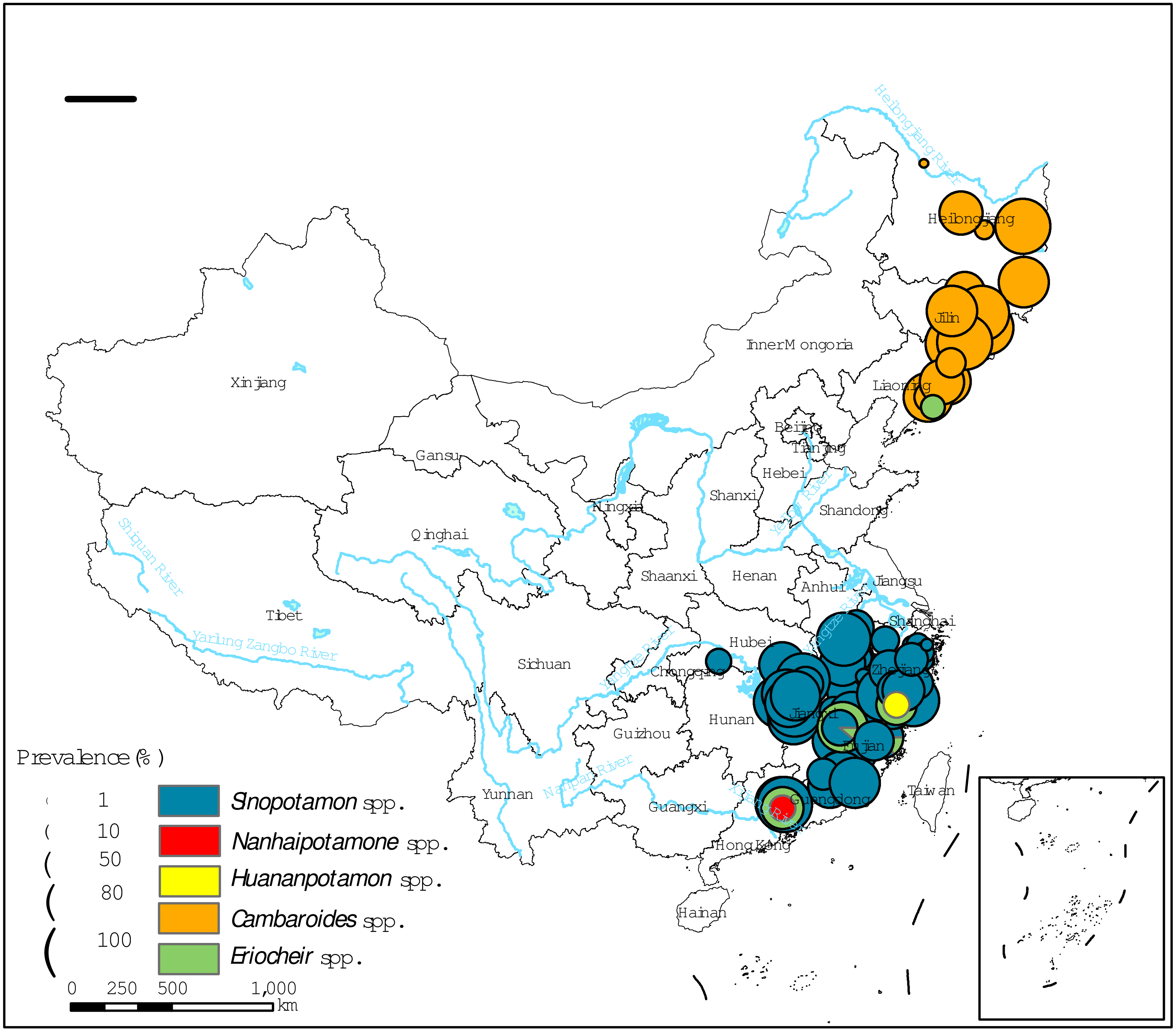

Fig 4b. Spatial distribution of P. skrjabini infection in the second intermediate hosts.

Click here to access/download;Figure;Fig 4b.eps

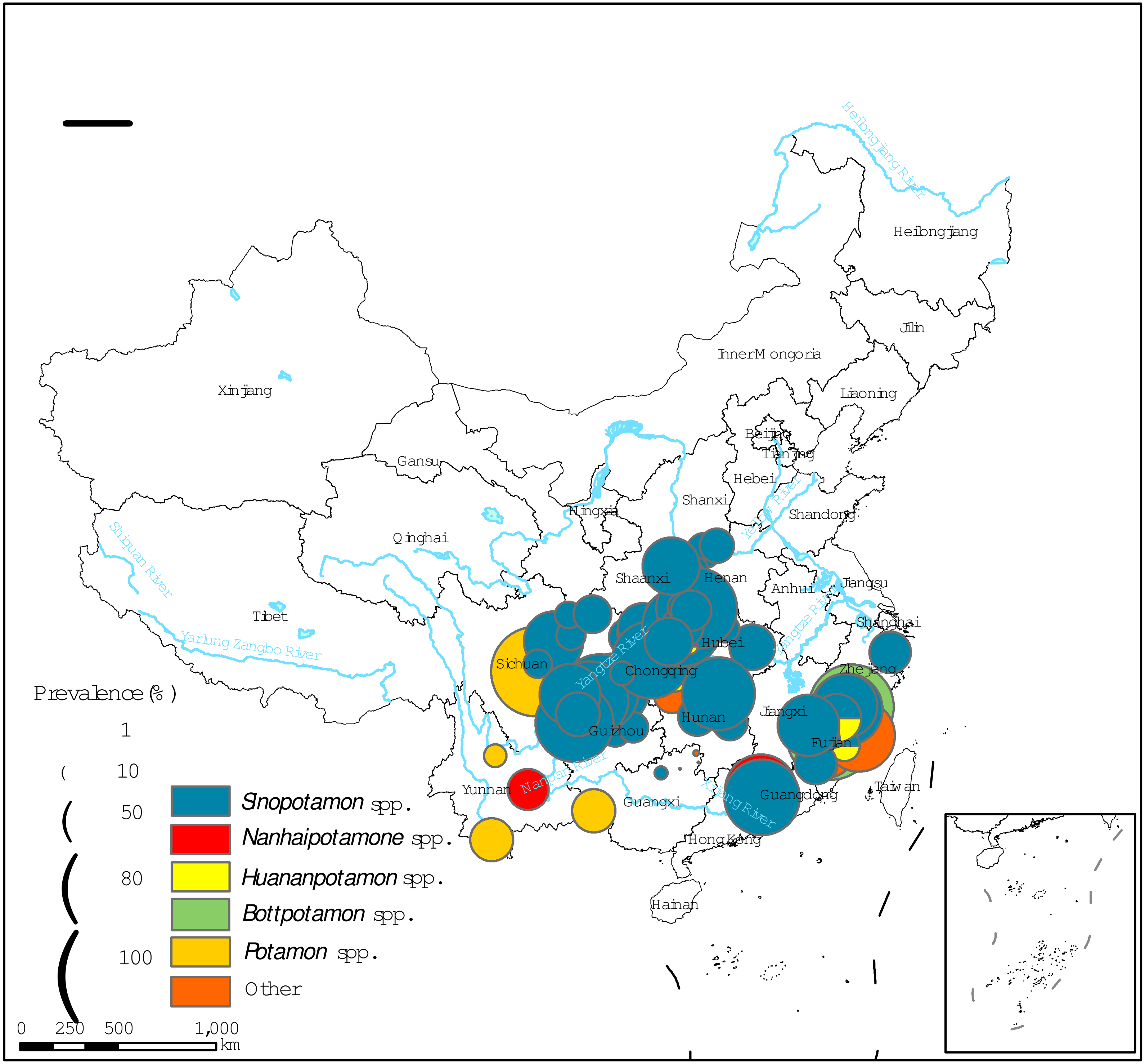

Fig 5. Environmental characteristics of areas with reported Paragonimus infections in China.

Click here to access/download;Figure;Fig 5.tif

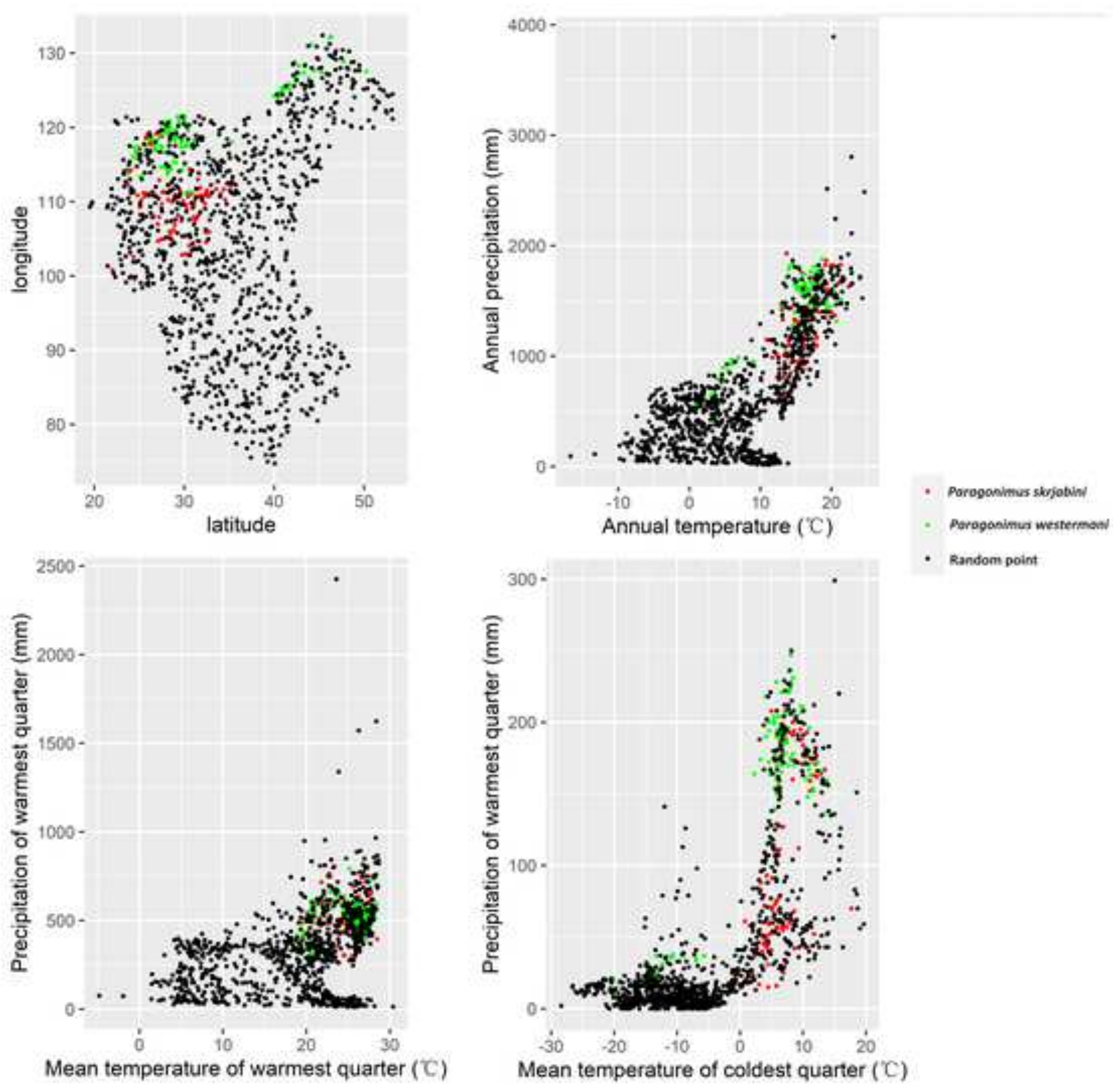

S1a Fig. Forest plots of prevalence of Paragonimus in humans.

Click here to access/download
Supporting Information
S1a_Fig.tif

S1b Fig. Forest plots of prevalence of P. westermani in the first intermediate host.

Click here to access/download
Supporting Information
S1b_Fig.tif

S1c Fig. Forest plots of prevalence of P. skrjabini in the first intermediate host.

Click here to access/download
Supporting Information
S1c_Fig.tif

S1d Fig. Forest plots of prevalence of P. westermani in the second intermediate host.

Click here to access/download
Supporting Information
S1d_Fig.tif

S1e Fig. Forest plots of prevalence of P. skrjabini in the second intermediate host.

Click here to access/download
Supporting Information
S1e_Fig.tif

S1f Fig. Forest plots of prevalence of P. skrjabini in animal reservoir.

Click here to access/download
Supporting Information
S1f_Fig.tif

S1g Fig. Forest plots of prevalence of P. westermani in animal reservoir.

Click here to access/download
Supporting Information
S1g_Fig.tif

S2a Fig. Funnel plot for assessing publication bias in studies reporting prevalence of Paragonimus in humans.

Click here to access/download
Supporting Information
S2a_Fig.tif

S2b Fig. Funnel plot for assessing publication bias in studies reporting prevalence of P. skrjabini in the first intermediate host.

Click here to access/download
Supporting Information
S2b_Fig.tif

S2c Fig. Funnel plot for assessing publication bias in studies reporting prevalence of P. westermani in the first intermediate

Click here to access/download
Supporting Information
S2c_Fig.tif

S2d Fig. Funnel plot for assessing publication bias in studies reporting prevalence of P. skrjabini in the second intermediate

Click here to access/download
Supporting Information
S2d_Fig.tif

S2e Fig. Funnel plot for assessing publication bias in studies reporting prevalence of P. westermani in the second intermediate

Click here to access/download
Supporting Information
S2e_Fig.tif

S2f Fig. Funnel plot for assessing publication bias in studies reporting prevalence of P. skrjabini in animal reservoir.

Click here to access/download
Supporting Information
S2f_Fig.tif

S2g Fig. Funnel plot for assessing publication bias in studies reporting prevalence of P. westermani in animal reservoir.

Click here to access/download
Supporting Information
S2g_Fig.tif

Click here to access/download
Supporting Information - Compressed/ZIP File Archive
S1-S10 Tables.zip
