# Supplementary material for: Infection and biogeographical characteristics of Paragonimus westermani and P. skrjabini in humans and animal hosts in China: A systematic review and meta-analysis
Source: PLoS Negl Trop Dis. 2024 Aug 5;18(8):e0012366. doi: 10.1371/journal.pntd.0012366 (PMC11326572; doi:10.1371/journal.pntd.0012366)
Supplement: Attachment — Submitted filename: Response to Reviewers.doc [file pntd.0012366.s016.doc]

**Reviewer #1**

**Summary and General Comments**

**Point 1:**

This study provides a comprehensive meta-analysis for studies on the epidemiology paragonimiasis in China. I would suggest that the authors add in the Introduction some information about the possible socio-economic impacts of the disease in endemic areas.

**Response to point 1:** Thank you for your acknowledgment and suggestion. We have supplemented the impact of *Paragonimus* infection on the socioeconomic aspects as “Misdiagnosis of paragonimiasis as pulmonary tuberculosis or lung cancer can lead to significant socioeconomic losses and impose a mental and physical burden on patients due to unnecessary hospitalization, laboratory tests, surgical procedures, and prolonged medication” (please see lines 85-88).

**Point 2:**

In the discussion: is there any explanation for the marked gender difference in the prevalence of the infection?

**Response to point 2:** The explanation for gender differences in infection rates has been added to the discussion section, such as “The number of cases in males is significantly higher than in females. This disparity is attributed to differences in behavior and occupational exposure. There is a higher proportion of males among fishermen, and males are more likely than females to catch and consume freshwater crabs and crayfish” (please see lines 428-432).

**Point 3:** Based on this extensive review I think it would be helpful to add any suggestions to standardize future surveys.

**Response to point 3:** Thank you for your suggestion. We have added recommendations and prospects for future research in the conclusion section of the manuscript, such as “In the future, it is advisable to incorporate paragonimiasis into China's China's National Notifiable Infectious Diseases Surveillance System to comprehensively monitor the incidence of the disease and identify high-risk populations more accurately. Furthermore, it is essential to systematically investigate the prevalence of *Paragonimus* spp. in various hosts in endemic areas and analyze the factors influencing these rates to enhance our understanding of the dynamics of Paragonimus spp. transmission.” (please see lines 517-523).

**Reviewer #2**

**Methods**

**Point 1:** The work complies with the aspects mentioned above. The period covered by the study, although the end date is stated (it is understood from 2010 to the present), the beginning could be better specified (it is a very broad period to say before 1990)

**Response to point 1:** Thank you very much for your reminder. The earliest study included in our analysis was conducted in 1954, and we have added this in Lines 226-227 and made corrections in the tables within the manuscript.

**Results**

**Point 2:** Line 202-203: The total number of publications is not clear (876 - 10642?)

**Response to point 2:** We apologize for the error at this point. We have corrected 876 to 16,876, please see Line 220.

**Point 3:** Line 222-224: Table 1 does not show human cases in Hong Kong and Macau either.

**Response to point 3:** Thank you for your reminder. We have made the necessary additions in Lines 251-252.

**Point 4:** Line 227: 71.12% or 71.21%

**Response to point 4:** Thank you for your review. We have corrected "71.12%" to "71.21%" in Line 255.

**Point 5:** Fig 2: the reference system with parentheses is difficult to interpret

**Response to point 5:** I apologize for any confusion regarding the legend. I have corrected the issue and re-uploaded all images. The base color of the map represents the number of cases in each province, while the size of the circles represents the number of cases at each survey point.

**Point 6:** Line 219-222: it could be indicated that the cases by gender, source and age are obviously above those documented, explicitly consider that there is a significant number that is not specified

**Response to point 6:** Despite some studies providing detailed information on the gender, age, and source of cases, most studies only report the total number of cases. Therefore, the number of cases by gender, source, and age is lower than those documented, we have added this information to lines 248-250 of the manuscript as per your suggestion.

**Point 7:** Fig 1: the value 261 is not clear? (38+107+174+58=377)

**Response to point 7:** I apologize for the misunderstanding. This was because, among the 261 included studies, some contained data on two or more types of hosts (e.g., a single article investigating humans, intermediate hosts, and animal reservoirs simultaneously).

To avoid any misunderstanding, we have added a note below the title of Figure 1 (Due to some studies simultaneously investigating humans, intermediate hosts, and animal reservoirs, the total number of literature included in the systematic review (271) does not equal the sum of literature for different types of hosts.), Please see lines 230-232.

**Point 8:** Line 297: 94 estudios and line 307: 81 estudios = 175. En la line 206 menciona 172 en second intermediate host

**Response to point 8:** I apologize for the misunderstanding. Similarly, several studies reported infection rates of both lung flukes in crustaceans, resulting in the total number of literature included in the study for the second intermediate host (172) being less than the sum of literature for *P. westermani* and *P. skrjabini*. (see Lines 232-235)

**Summary and General Comments**

The work is a valuable contribution by summarizing the situation of infection by *P. westermani* and *P. skrjabini* in their main hosts, as well as the geographical distribution. The figures that allow a visual-spatial representation of the infection situation in China stand out, being a fundamental pillar when establishing prevention-control measures.

We greatly appreciate your recognition.

**Point 9:** When only Paragonimus is mentioned without specifying the species, it could be indicated as Paragonimus spp.

**Response to point 9:** Thank you very much for your suggestion. We have carefully reviewed the manuscript and replaced "*Paragonimus*" which does not specifically refer to *P. westermani* and *P. skrjabini* with "*Paragonimus* spp." Please check the Abstract and Introduction sections.

**Point 10:** Line 87: P. westerman would be *P. westermani*

**Response to point 10:** Thank you for your reminder. We have corrected it in Line 99.

**Point 11:** Lines 87 - 91: would include some bibliographical reference

**Response to point 11:** Thank you for your reminder. We have cited the reference in Line 103.

**Point 12:** Line 446: ...prevalence of 52.02 % (95% CI 42.65 - 75.79%). The CI differs from Table 4

**Response to point 12:** Thank you for your correction. We have revised the confidence intervals (Please see line 478).

**Point 13:** Line 458: P. iloktsuensis or P. iloktsuenensis?*T. chiui*: is it previously defined?

**Response to point 13:** Thank you for your correction. We have corrected *P. iloktsuensis* to *P. iloktsuenensis* and changed *T. chiui* to *Tricula chiui* (Please see line 490).

**Reviewer #3**

**Abstract**

**Point 1:** Line 36 Which are they? (Which electronic databases were used for literature retrieval?)

**Response to point 1:** Thank you for your suggestion. The eight databases included are China National Knowledge Infrastructure (CNKI), Chinese Wanfang Database (CWFD), Chongqing VIP, SinoMed, Medline, Embase, PubMed, and Web of Science. This information has already been detailed in the Methods section of the manuscript. Additionally, as per your suggestion, we have included this information in the abstract. Please see Lines 36-37.

**Point 2:** Line 57 and low latitude? (Could altitude also be considered as one of the influencing factors?)

**Response to point 2:** Thank you for your suggestion. In addition to temperature and precipitation, altitude is also an important factor influencing the distribution of the two species of *Paragonimus*. We have added this information to the abstract in Line 56.

**Point 3:** Line 59 and keywords (Lack of keywords)

**Response to point 3:** Per your reminder, we have added the keywords in Lines 59-61.

**Point 4:** Line 92 reference?

**Response to point 4:** We have added the references in Line 104.

**Methods**

**Point 5:** Line 140 prevalence?? (Should infection rate be prevalence?)

**Response to point 5:** Thank you for your reminder, we have followed the advice and changed “positive rate” to “prevalence”.

**Point 6:** Line145 pooled prevalence and CI (How are pooled prevalence and confidence intervals (*CI*) calculated?)

**Response to point 6:** For some studies, not all participants underwent etiological detection. Instead, skin testing was performed first, followed by etiological detection for those who tested positive. For these studies, we used the total number of participants as the denominator and the number of etiological-positive cases as the numerator to estimate the pooled prevalence and *CI*. Based on your suggestion, we have added the specific calculation method (Peto) and explanation in the statistical methods section on Lines 172-174.

**Point 7:** Line 153 prevalence?? (Is prevalence appropriate?)

**Response to point 7:** We have corrected infection rates to prevalence in the manuscript.

**Results**

**Point 8:** Line 202 Analyzing the sentence of less than a thousand falls	but of ten thousand

**Response to point 8:** We apologize for the error at this point. We have corrected 876 to 16,876, please see Line 220.

**Point 9:** Line 242 how to calculate the *CI*, without reference to materials and methods

**Response to point 9:** Thank you for your suggestion, for the confidence interval (*CI*), we have added the specific calculation method (Peto) and explanation in the statistical methods section on Lines 172-174.

**Point 10:** Line244 Check that materials and methods are adequately explained

**Response to point 10:** Thank you for your suggestion, we have added an explanation of the meta-regression model in Lines 176-178.

**Point 11:** Lines 247, 285, 326, and 353 Several of these important variables were not adequately marked and explained in materials and methods

**Response to point 11:** Thank you very much for your suggestion. We have added the important variables that were overlooked in the methodology. Please refer to Lines 152-155.

**Point 12:** Line 270 Separate… (Missing space here)

**Response to point 12:** We apologize for the missing space here. It has been corrected in Line 298.

**Conclusions**

**Point 13:** Line 492 low altitude (Could altitude also be considered as one of the influencing factors?)

**Response to point 13:** As you pointed out, altitude is also one of the important factors influencing the distribution of *P. westermani* and *P. skrjabini*. We have added this information in Line 530.
